# The International Collaborative Animal Study of mobile phone radiofrequency radiation carcinogenicity and genotoxicity: the Japanese study

**DOI:** 10.1093/toxsci/kfag002

**Published:** 2026-01-12

**Authors:** Katsumi Imaida, Mayumi Kawabe, Jianqing Wang, Masanao Yokohira, Norio Imai, Kang-Hyun Han, Yong-Bum Kim, Sang Bong Jeon, Hye Sun Kim, Young Hwan Ahn

**Affiliations:** Kagawa University, Takamatsu, Kagawa 760-8521, Japan; DIMS Institute of Medical Science, Inc., Ichinomiya, Aichi 491-0113, Japan; IWATA Research Institute, Pathology Lab. Aichi Branch, Trans Genic Inc., Iwata, Shizuoka 437-1213, Japan; Department of Molecular Oncologic Pathology, Faculty of Medicine, Kagawa University, Miki, Kagawa 761-0793, Japan; Department of Electrical and Mechanical Engineering, Nagoya Institute of Technology, Nagoya, Aichi 466-8555, Japan; Department of Medical Education, Faculty of Medicine, Kagawa University, Miki, Kagawa, 761-0793, Japan; DIMS Institute of Medical Science, Inc., Ichinomiya, Aichi 491-0113, Japan; Research Department, Nihon Bioresearch Inc., Hashima, Gifu 501-6251, Japan; Division of Next Generation Non-Clinical Research, Korea Institute of Toxicology, Daejeon 34114, Republic of Korea; Division of Next Generation Non-Clinical Research, Korea Institute of Toxicology, Daejeon 34114, Republic of Korea; Radio Research Division, Electronics and Telecommunications Research Institute (ETRI), Daejeon 34129, Republic of Korea; Department of Neurosurgery, Ajou University School of Medicine, Suwon 16499, Republic of Korea; Department of Neurosurgery, Ajou University School of Medicine, Suwon 16499, Republic of Korea; Neuroscience Graduate Program, Department of Biomedical Sciences, Graduate School of Ajou University, Suwon 16499, Republic of Korea

**Keywords:** CDMA 900 MHz RF-EMF, long-term carcinogenicity bioassay, genotoxicity, NTP validation study, international collaborative study

## Abstract

The potential carcinogenic and genotoxic effects of radiofrequency electromagnetic fields, particularly those emitted by mobile communication systems, have raised public health concerns. A previous study by the U.S. National Toxicology Program suggested increased incidences of gliomas and cardiac schwannomas in rats exposed to high levels of RF radiation. To evaluate these findings, an international collaborative study was initiated between Japan and Korea. Male Hsd:Sprague Dawley SD rats were exposed to 900 MHz CDMA-modulated RF-EMFs at a whole-body specific absorption rate of 4 W/kg for 18 h and 20 min daily over 2 yr. The study included a 28-d preliminary toxicity study, genotoxicity assays (alkaline comet and micronucleus tests), and a 2-yr carcinogenicity assessment. All procedures followed OECD guidelines and Good Laboratory Practice. No statistically significant increases in the incidences of neoplastic or non-neoplastic lesions were found in any major organ, including the brain, heart, and adrenal glands. Genotoxicity assays revealed no evidence of DNA damage or chromosomal aberrations in RF-exposed rats. A higher survival rate in the RF-exposed group, likely due to lower body weight and food consumption, was observed. This study, performed in Japan, jointly planned and executed by Japan and Korea, provides strong evidence that long-term exposure to 900 MHz RF-EMFs did not produce reproducible carcinogenic or genotoxic effects in male rats. Combined with data from the Korean counterpart study, these results are expected to contribute to future international assessments of the carcinogenic potential of electromagnetic radiation.

The widespread use of wireless communication devices has raised public health concerns about potential carcinogenic and genotoxic effects of radiofrequency electromagnetic fields (RF-EMFs). As wireless technology becomes increasingly integrated into daily life, clarifying the long-term biological impact of chronic RF-EMF exposure is a priority for science and regulatory policy.

Epidemiological studies, including the INTERPHONE and Danish cohort studies, have examined possible associations between mobile phone use and tumors such as glioma and acoustic neuroma, but have not provided consistent evidence for causality (Frei et al. 2011; Kundi 2012). Based on limited evidence in humans and experimental animals, the International Agency for Research on Cancer (IARC) classified RF-EMFs as “possibly carcinogenic to humans” (Group 2B) (IARC Working Group on the Evaluation of Carcinogenic Risks to Humans 2013). This precautionary designation remains controversial due to uncertainties in exposure assessment and interpretation of experimental findings, underscoring the need for robust, reproducible animal studies.

To address this, the U.S. National Toxicology Program (NTP) conducted 2-yr studies in Hsd:Sprague Dawley SD rats and B6C3F1/N mice exposed to 900 MHz CDMA- or GSM-modulated RF-EMFs (Wyde et al. 2016; NTP 2018a, 2018b). In male rats, high-level exposure was associated with increased incidences of malignant cardiac schwannomas and brain gliomas. Although these associations were statistically marginal and of uncertain biological significance, they stimulated renewed scientific and regulatory interest.

This international collaborative study was designed to evaluate the findings of the NTP radiofrequency bioassay under harmonized experimental conditions. This collaboration involved joint planning, synchronized execution, and harmonized evaluation criteria over a 5-yr period. The present Japanese study, conducted in accordance with Good Laboratory Practice and OECD guidelines, exposed male Hsd:Sprague Dawley SD rats to 900 MHz CDMA-modulated RF-EMFs at a whole-body specific absorption rate (SAR) of 4 W/kg for 2 yr to assess carcinogenicity and for 14 wk to evaluate genotoxicity.

The genotoxic potential of RF-EMFs has been widely examined using in vitro and in vivo methods, including the micronucleus test, comet assay, chromosomal aberration analysis, and γ-H2AX foci detection (McNamee et al. 2003; Markova et al. 2005; Kennedy et al. 2012). Some in vitro studies reported RF-induced DNA damage or micronucleus formation (Diem et al. 2005; Zeni et al. 2005), but well-conducted in vivo studies—particularly those adhering to OECD guidelines—have generally not demonstrated such effects (Shirai et al. 2005; Verschaeve et al. 2010; Smith-Roe et al. 2020). Accordingly, the present study incorporated an alkaline comet assay and a micronucleus test to detect potential DNA or chromosomal damage in multiple tissues after subchronic RF-EMF exposure.

Interpretation of long-term rodent carcinogenicity data for spontaneous tumors such as gliomas and cardiac schwannomas requires caution, as these are typically late-onset and occur at low background incidence (Lee et al. 2010; Weber et al. 2011; Bertrand et al. 2014; Sacaan et al. 2019). Differences in survival between control and exposed groups, especially low late survival in control groups, can markedly influence observed tumor rates, independent of any exposure effect. Moreover, glioma “not otherwise specified” (NOS) in rats is pathologically distinct from human glioblastoma multiforme and is considered by several expert pathology groups to be a rat-specific lesion with limited relevance for human cancer risk assessment (Bradley et al. 2020).

This study comprehensively evaluates the carcinogenic and genotoxic potential of long-term RF-EMF exposure under validated experimental conditions. Endpoints include reproductive outcomes (Table 1), neoplastic lesions (Table 2), non-neoplastic histopathology (Table 3), and genotoxicity assays (Table 4: alkaline comet assay; Table 5: micronucleus test). By applying rigorous design, execution, and peer-reviewed pathology assessment, it aims to clarify the reproducibility of the NTP findings and contribute to the evidence base guiding public health decisions on RF-EMF exposure.

**Table 1.** Reproductive findings of the F0 females in the 2-yr carcinogenicity study.

	Cage control	Sham-exposed	RF-exposed
Parameters	0 W/kg	0 W/kg	4 W/kg
	*n* = 30	*n* = 30	*n* = 30
Gestation period (days)	23.4 ± 0.5	23.6 ± 0.6	23.3 ± 0.5
Delivery index^a^ (%)	93	93	90
No. of delivered per litter	13.5 ± 2.4	12.1 ± 3.3	12.7 ± 2.9
No. of live pups per litter	13.2 ± 2.6	11.7 ± 3.1	12.5 ± 3.0
No. of dead pups per litter	0.3 ± 0.7	0.4 ± 0.8	0.2 ± 0.8
Sex ratio^b^ (%)	55.3 ± 18.5	49.4 ± 14.1	53.6 ± 15.2

^a^ (No. of F_0_ rats with live pups/No. of pregnancies) × 100.

^b^ (No. of live male pups/No. of live pups) × 100.

No statistically significant differences in delivery index were observed among the groups by Fisher’s exact test.

**Table 2.** Neoplastic findings in the main target organs in the 2-yr carcinogenicity study.

		Cage control	Sham-exposed	RF-exposed
Organ	Findings	0 W/kg	0 W/kg	4 W/kg
		*n* = 70	*n* = 70	*n* = 68
Heart	Schwannoma, endocardial	1 (1%)	1 (1%)	1 (1%)
Brain	Tumor, granular cell, benign	0	3 (4%)	0
	Glioma, NOS	2 (3%)	0	1 (1%)
	Tumor, granular cell, malignant	1 (1%)	0	0
Adrenal glands	Adenoma, cortex	0	3 (4%)	1 (1%)
	Pheochromocytoma, benign	10 (14%)	7 (10%)	9 (13%)
	Carcinoma, cortex	1 (1%)	0	1 (1%)
	Pheochromocytoma, malignant	4 (6%)	1 (1%)	0
	Pheochromocytoma, complex, malignant	0	0	1 (1%)

No statistically significant differences in any tumor incidence were observed among the groups by Peto’s test.

**Table 3.** Non-neoplastic findings in the main target organs in the 2-yr carcinogenicity study.

		Cage control	Sham-exposed	RF-exposed
Organ	Findings	0 W/kg	0 W/kg	4 W/kg
		*n* = 70	*n* = 70	*n* = 68
Heart	Thrombus	7 (10%)	2 (3%)	0*
	Rodent progressive cardiomyopathy	63 (90%)	61 (87%)	56 (82%)
	Mineralization, myocardium	1 (1%)	2 (3%)	0
	Mineralization, media/wall artery	13 (19%)	6 (9%)	3 (4%)*
	Infiltrate, inflammatory cell	1 (1%)	0	1 (1%)
	Polyarteritis	6 (9%)	0*	0*
	Hyperplasia, Schwann cell, subendocardium	0	1 (1%)	2 (3%)
	Hyperplasia, mesothelium, epicardium	2 (3%)	3 (4%)	0
Brain	Infiltrate, inflammatory cell, perivascular	2 (3%)	0	0
	Aggregation, granular cell	0	0	1 (1%)
Adrenal glands	Thrombus	1 (1%)	1 (1%)	0
	Atrophy, cortex	0	0	1 (1%)
	Degeneration, cystic	1 (1%)	0	0
	Vacuolation, cortex, decreased, focal	5 (7%)	6 (9%)	3 (4%)
	Vacuolation, cortex, increased, diffuse	1 (1%)	1 (1%)	1 (1%)
	Vacuolation, cortex, increased, focal	12 (17%)	7 (10%)	18 (26%)^†^
	Polyarteritis	3 (4%)	4 (6%)	0
	Hypertrophy, cortex	17 (24%)	23 (33%)	23 (34%)
	Hyperplasia, cortex	42 (60%)	38 (54%)	28 (41%)*
	Hyperplasia, medulla	26 (37%)	34 (49%)	17 (25%)^††^

Significantly different from the cage control group; *P < 0.05 (Fisher’s exact test).

Significantly different from the sham-exposed group; ^†^*P* < 0.05, ^†^^†^*P* < 0.01 (Fisher’s exact test).

**Table 4.** Alkaline comet assay.

Groups	No. of rats	% tail DNA				
		Liver	Peripheral blood	Cerebellum	Hippocampus	Frontal cortex
Cage control	5	1.92 ± 1.26	0.81 ± 0.05	1.45 ± 0.32	2.76 ± 1.31	2.61 ± 0.63
Sham-exposed	5	1.83 ± 0.99	0.79 ± 0.07	1.42 ± 0.36	2.68 ± 0.88	2.61 ± 0.57
RF-exposed	5	1.59 ± 0.74	0.85 ± 0.06	1.47 ± 0.34	1.99 ± 0.33	2.50 ± 0.69
Positive control^a^	3	29.30 ± 3.88**	24.83 ± 1.74*	30.52 ± 0.99**	34.65 ± 3.84**	35.84 ± 2.10*

a Ethyl methanesulfonate 200 mg/kg.

Significantly different from the cage control group; *P < 0.05, **P < 0.01 (Student’s t-test).

**Table 5.** Micronucleus test.

Groups	No. of rats	MNIEs (%)	IEs (%)
Cage control	5	0.080 ± 0.021	39.4 ± 4.9
Sham-exposed	5	0.080 ± 0.033	41.6 ± 3.9
RF-exposed	5	0.080 ± 0.041	40.7 ± 5.0
Positive control^a^	5	0.540 ± 0.123*	34.8 ± 6.7

^a^ Ethyl methanesulfonate 200 mg/kg.

MNIEs (%): Micronucleated immature erythrocytes (MNIE)/Immature erythrocytes (IE).

IEs (%): Immature erythrocytes (IE)/Erythrocytes.

Significantly different from the cage control group; *P < 0.05 (Student’s t-test).

## Materials and methods

This study, conducted in collaboration with Korean researchers, aimed to evaluate the findings of the U.S. National Toxicology Program (NTP) regarding the potential carcinogenicity of radiofrequency radiation (RFR) exposure in rats. Common experimental methods and animal models were adopted by both Japan and Korea to ensure consistency and comparability of results. The overall study design—including exposure frequency, modulation, SAR level, and animal strain—was developed in close consultation between the Japanese and Korean research teams. The international advisory committee reviewed and endorsed the harmonized protocol to ensure comparability across the 2 studies while taking into account the technical feasibility at each facility.

The RFR exposure system utilized custom-developed reverberation chambers, a joint development between Nagoya Institute of Technology and Korea Electronics and Telecommunications Research Institute (Jeon et al. 2021; Ito et al. 2022). The exposure frequency was set at 900 MHz with CDMA modulation, mimicking the signals used by mobile phones. The exposure level for rats was established at a whole-body average specific absorption rate (SAR) of 4 W/kg. This level was selected because, in the previous NTP study, the maximum exposure level of 6 W/kg increased the rats’ deep body temperature—even though the rise was kept below 1 °C—in a manner that could still potentially influence tumor development.

**Fig. 1.**
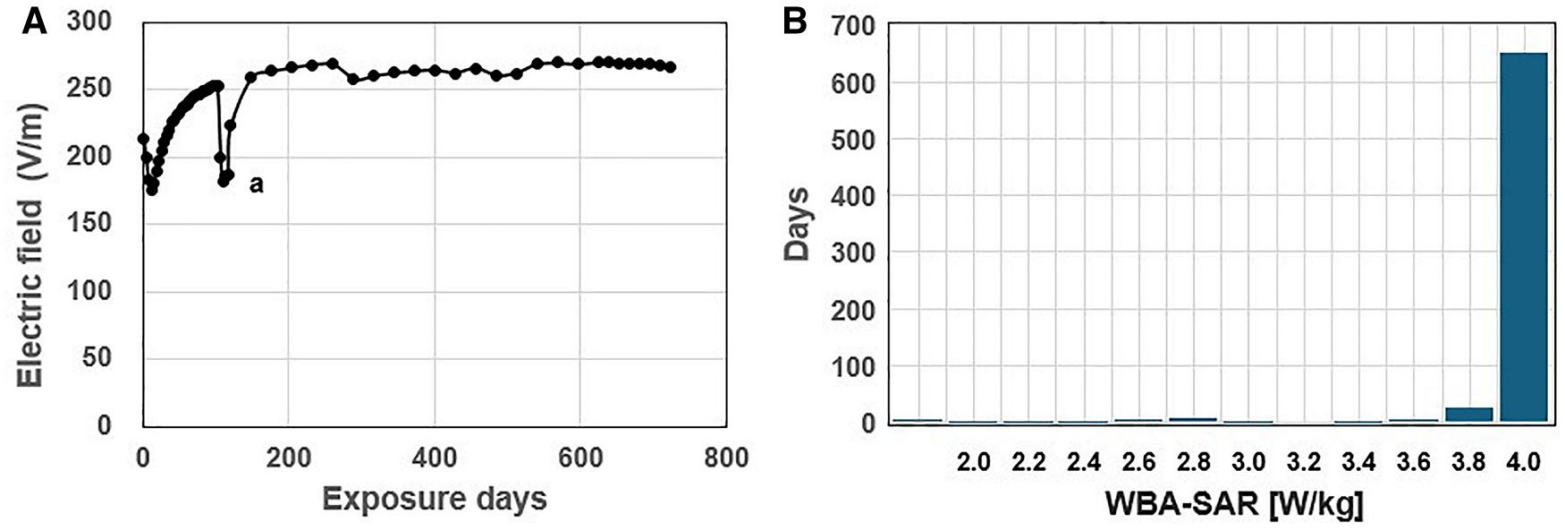
RF exposure levels during the study period. A) Average daily electric field level required to produce a whole-body average SAR of 4 W/kg. a) The electric field levels on the annotated days (approximately 4 wk) only produced a whole-body average SAR of approximately 2-3 W/kg due to amplifier malfunction. B) Histogram of day-average whole-body average SAR.

RFR exposure was conducted for 18 h and 20 min per day, structured as 2 periods (approximately 12:00 to 15:00 and 16:40 to 08:00) with 10-min on/off cycles. The exposure level was managed by monitoring and controlling the mean electric field level in the reverberation chamber, ie, the mean electric field level was monitored every minute with an electric field probe and was controlled by PC software to be within ±3% of the electric field level to achieve the whole-body average SAR of 4 W/kg. A quantitative relationship between the mean electric field level in the reverberation chamber and the whole-body average SAR of 4 W/kg was established based on a large-scale finite difference time domain (FDTD) simulation. The derived quantitative relationship between the mean electric field level and the whole-body average SAR of 4 W/kg is a 2-dimensional approximation model which incorporates both the body mass and the number of rats, and was validated through measurements involving 80 live rats. Using the approximation model, throughout the 2-yr study, the whole-body average SAR was successfully maintained at 4 W/kg for 94% of the total duration, with exceptions for approximately 4 wk due to a power amplifier malfunction. During the power amplifier malfunction, only one of the 2 amplifiers was operational, so the whole-body average SAR was maintained at approximately half the target value, 2 W/kg, as shown in Fig. 1.

### Animals and husbandry

The 28-d preliminary study used Hsd:Sprague Dawley SD rats imported from Envigo (Indianapolis, USA) to harmonize major experimental conditions with those of the NTP study, although the present study was not intended as a direct replication. All studies were conducted in compliance with applicable Good Laboratory Practice (GLP) standards and animal welfare guidelines, including Japanese laws such as the “Act on Welfare and Management of Animals” and “Standards relating to the Care and Keeping and Reducing Pain of Laboratory Animals,” as well as guidelines from the Organization for Economic Co-operation and Development (OECD), specifically TG451 for Carcinogenicity Studies, TG489 for in vivo Mammalian Alkaline Comet Assay, TG474 for Mammalian Erythrocyte Micronucleus Test, and TG407 for Repeated Dose 28-Day Oral Toxicity Study in Rodents.

Animals were housed in rooms with appropriate biological containment. Housing conditions included controlled temperature (23 ± 3 °C) and relative humidity (55 ± 15%). A 12-h light/dark cycle was maintained (7:00 to 19:00), with a ventilation rate of 10 or more air changes per hour. Animals were housed individually after weaning, whereas during the lactation period, dams and pups were kept together in one cage. Cage changes occurred at least once per week.

Two types of cages were utilized: plastic cages (W257 × D426 × H200 mm) with stainless steel lids for the cage control group, and polycarbonate cages (W235 × D260 × H210 mm) for the RF-exposed and sham-exposed groups. Aspen chips (TAPVEI) were used as bedding, and M-bricks (TAPVEI) were provided for environmental enrichment. Contaminant levels in bedding and enrichment were confirmed to be below maximum permissible concentrations. Feed, PicoLab Rodent Diet 20 (Code No. 5053) (PMI Nutrition International, LLC) was provided ad libitum. During the 2-yr carcinogenicity study, the animals were fed Teklad Global Rodent Diet (code no. 2914; ENVIGO International Holdings Inc.), a low-protein formulation routinely used in long-term carcinogenicity studies, including those conducted by NTP. The diet was provided ad libitum. Because standard-protein diets have been associated with increased mortality in long-term studies, this low-protein diet was used throughout the 2-yr study (Keenan et al. 1994). Cage controls received feed in feeders attached to the cage lid, whereas RF-exposed and sham-exposed animals received feed in ceramic feeders placed inside the cages. Contaminants in the feed were also monitored and confirmed to be below acceptable levels. Drinking water was tap water from Ichinomiya City. For RF-exposed and sham-exposed groups, water was sterilized using a UV flow sterilization device and supplied via an automatic watering system, with water replaced every 3 h. Water quality was periodically checked to ensure compliance with standards.

To ensure uniform RF exposure, cage rotation was systematically performed. Within the exposure chambers, racks were rotated left and right every 2 wk. Cages were arranged according to a predefined layout and rotated vertically (top to bottom) once a week. Additionally, horizontal cage rotations were performed periodically (eg, every 13 wk from week 14 to week 66) in 2-yr carcinogenicity study. From week 69 onwards in the 2-yr study, front-to-back cage rotations within racks were also implemented every 2 wk. Individual animals were identified using oil ink on the tail for dams and early pups, and by ear cuts/punches for pups after weaning. Monitoring animals were included in both studies to confirm normal health, with body weight and general condition observed, and necropsy performed at scheduled sacrifice. Microbiological monitoring was conducted every 6 mo for the 2-yr study, showing no abnormalities.

### 28-Day preliminary study

**Fig. 2.**
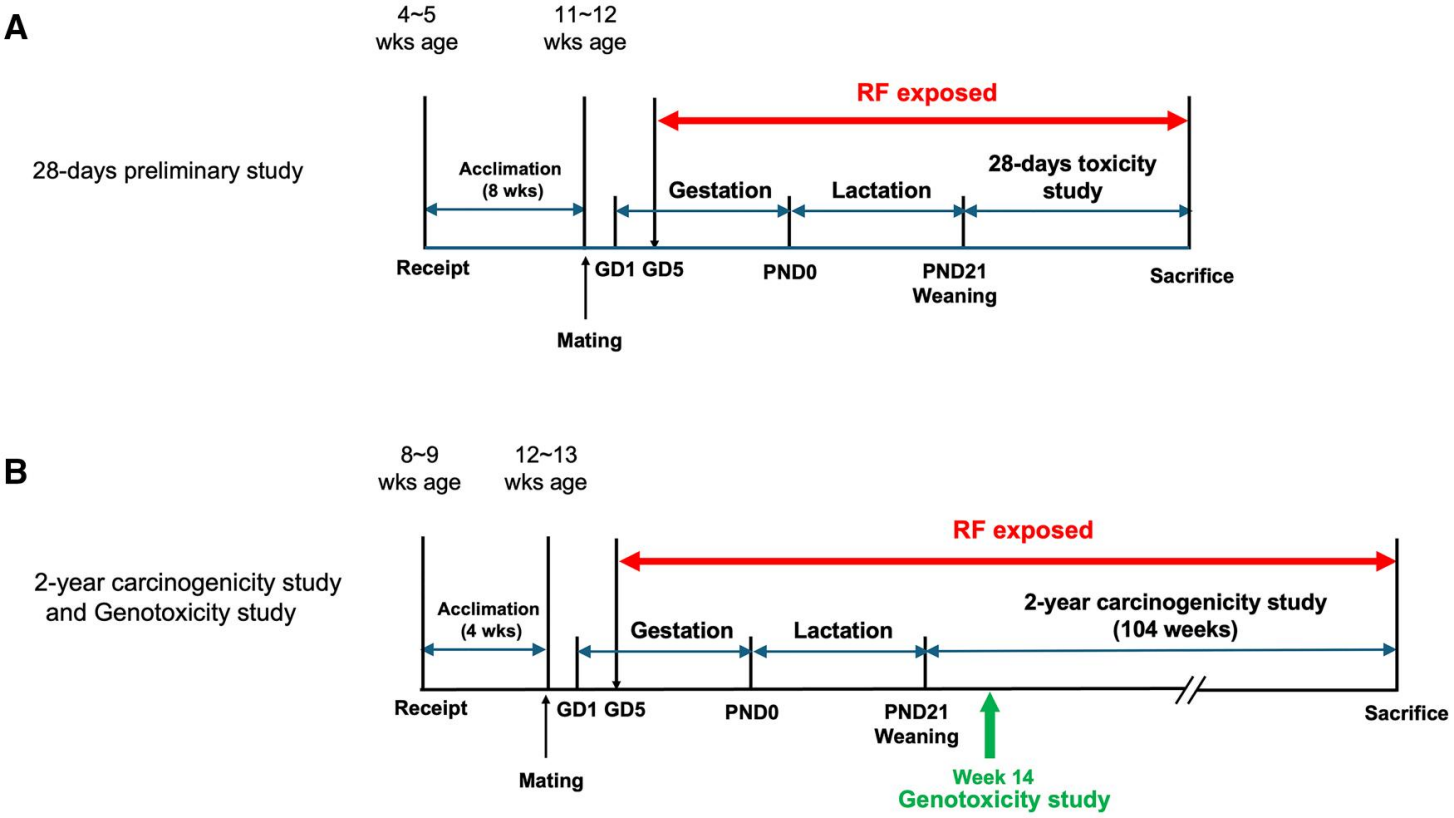
Experimental protocols for the 28-d preliminary, 2-yr carcinogenicity, and genotoxicity studies. RF exposure was initiated on gestation day (GD) 5 in F0 rats and continued through the lactation period until postnatal day (PND) 21. After weaning, RF exposure was continued in F1 offspring for 28 d [(A) preliminary study], 14 wk [(B) genotoxicity study], or 2 yr [(B) carcinogenicity study], depending on the experimental group.

This preliminary study was conducted to confirm the feasibility and safety of the 4 W/kg RF exposure level for the subsequent 2-yr carcinogenicity study. The experimental protocol is shown in Fig. 2. Female and male Harlan SD rats were received at 4 to 5 wk of age and acclimated for 6 d. Mating began when animals reached 11 to 12 wk of age by co-housing 1 male with 2 females in the evening. Pregnancy was confirmed by the presence of sperm in vaginal smears the next morning, designated as gestation day 1 (GD1). Mated females with confirmed pregnancy were assigned to the RF-exposed, sham-exposed, and cage-control groups until the target number was reached 12 per group. Pups were born (postnatal day 0, PND0), and litter sizes were adjusted to 8 pups (primarily male) at PND4. To evaluate reproductive performance from mating to parturition, the gestation period, number of implantation sites, delivery index, live birth index, number of delivered per litter, number of live pups per litter, number of dead pups per litter, and sex ratio were calculated. Weaning occurred at PND21, with age adjustment based on the latest-born pups. The 28-d toxicity study commenced when 10 litters per group were weaned, with one heaviest male pup selected from each litter, resulting in 10 male pups per group. Dams in the RF-exposed group began exposure at GD5, and pups were exposed with their mothers until weaning. Eight dummy animals were included to maintain a total of 20 animals in the exposure chamber before pup weaning, thereby ensuring uniform exposure levels in the chamber.

The general condition of both dams and pups was observed twice daily. Body weights of dams were measured on GD1, 5, 9, 12, 15, 18, 21, and PND1, 4, 7, 14, 21. Pup body weights were recorded at PND1 (by sex per litter), PND4 (before and after culling), PND7, 14, 17, 21, and weekly after weaning. Food consumption for dams was measured for various intervals during pregnancy and lactation, and weekly for pups. Body temperature was measured rectally at the end of afternoon exposure (around 15:00) on specific days for dams (GD5, 7, 11, 16, and PND1, 4, 7, 14) and pups (day 8, 16, 20, 27 after weaning). At the end of the 28-d exposure period, hematological (eg, RBC, HGB, WBC, differential counts) and blood biochemical parameters (eg, AST, ALT, BUN, T-CHO, ACTH) were measured in all surviving pups.

Pathological examinations for the 28-d preliminary study included necropsy of all surviving pups, with comprehensive observation of all organs and tissues. Specific organs and tissues were collected and preserved in 10% buffered formalin (with specific fixatives for testes and eyeballs). Bone marrow smears were prepared from the left femur for potential auxiliary diagnostic material. Organ weights (absolute and relative) were measured for key organs such as the brain, pituitary gland, heart, lungs, liver, spleen, kidneys, adrenal glands, testes, epididymides, prostate, seminal vesicles, salivary glands, thymus, and thyroid glands. Histopathological examination involved processing all collected organs and tissues into paraffin blocks, sectioning, and staining with Hematoxylin and Eosin (H&E) for microscopic observation. A bilateral pathology peer review between institutions was conducted by a toxicological pathologist from the Korea Institute of Toxicology (KIT).

### 2-Year carcinogenicity study

The main objective of this study was to evaluate the long-term carcinogenicity of 900 MHz CDMA RF exposure in rats, aiming to evaluate the reproducibility of the NTP study results. Experimental protocol is shown in Fig. 2. Male Harlan SD rats were used for the carcinogenicity evaluation. Animals were received at 8 to 9 wk of age and acclimated for 6 d. One male and one female rat were housed together in a single cage for mating in the evening when animals reached 12 to 13 wk of age, following similar procedures as the preliminary study to confirm pregnancy. Mated females with confirmed pregnancy were assigned to the RF-exposed, sham-exposed, and cage-control groups until the target number was reached, 30 per group. Litter sizes were adjusted to 8 pups (primarily male) at PND4. At weaning (PND21), 70 male pups per group were selected for the 2-yr carcinogenicity study, designated as Exposure Day 1. Pups in the RF-exposed group were exposed with their mothers until weaning. The exposure period for the carcinogenicity study lasted for 104 wk.

Reproductive findings of the F0 generation were meticulously recorded, including gestation period, number of implantation sites, delivery index, live birth index, number of delivered per litter, number of live pups per litter, number of dead pups per litter, and sex ratio.

General condition of the animals was observed twice daily throughout the study, including palpation every 4 wk at the time of body weight measurement. Body weights were measured twice weekly until week 17, then every 4 wk until week 86, and every 2 wk thereafter until necropsy. Food consumption was measured weekly until week 17, then every 4 wk until week 86, and every 2 wk thereafter until necropsy.

Pathological examinations were conducted on all animals. At the time of scheduled sacrifice, or upon death/euthanasia of moribund animals, necropsy was performed with comprehensive observation of all organs and tissues listed. Organs and tissues were collected and preserved in 10% buffered formalin, with specific fixatives used for testes and eyeballs. Bone marrow smears were prepared from the left femur for auxiliary diagnostic material.

For histopathological examination, all collected organs and tissues (including heart, spleen, lymph nodes, Peyer’s patch, thymus, pituitary gland, thyroid glands, parathyroid glands, adrenal glands, nasal cavity, trachea, lungs, salivary glands, pharynx, larynx, esophagus, stomach, small intestine, large intestine, liver, pancreas, kidneys, urinary bladder, testes, epididymides, prostate, seminal vesicles, preputial glands, mammary gland, brain, spinal cord, sciatic nerve, trigeminal nerve, aorta, eyeballs, Harderian glands, skin, bone and bone marrow, skeletal muscle, and macroscopic lesions) were embedded into paraffin blocks, sectioned, and stained with Hematoxylin and Eosin (H&E). Pathological diagnoses followed international standards such as INHAND, with the latest edition used for central nervous system lesions (Mann et al. 2012; Bradley et al. 2020). Non-neoplastic lesions were graded on a 4-point scale (Slight, Moderate, Marked, Severe). For specific brain cases, immunohistochemical staining was performed to confirm the diagnosis using the following antibodies: Vimentin (mouse monoclonal, 1:600; Dako, Denmark), Cytokeratin AE1/AE3 (mouse monoclonal, 1:1; Leica Biosystems, U.K.), S100 (rabbit polyclonal, 1:1600; Dako, Denmark), and Glial Fibrillary Acidic Protein (GFAP) (rabbit polyclonal, 1:1; Dako, Denmark).

Pathology peer review was a crucial component of the study. Initially, an institutional pathology peer review was conducted by a pathologist from Trans Genic Inc. A bilateral institutional pathology peer review was conducted between Japan and the Korea Institute of Toxicology (KIT) for all neoplastic lesions, as well as for specific organs of concern—including the brain, heart, and components of the nervous system (spinal cord, trigeminal nerve, and sciatic nerve) that showed notable findings in the NTP study. This review was performed in person at Kagawa University and KIT using virtual slides. Furthermore, an international third-party pathology peer review was implemented for all neoplastic and proliferative lesions in the heart and brain, involving reviewers from the USA, Korea, and Japan. This international third-party peer review was conducted via web conference, with virtual slides provided to the reviewers.

### Genotoxicity study

The genotoxicity study was conducted in parallel with the 2-yr carcinogenicity study rather than as an integrated component of it. Both studies proceeded concurrently from the beginning of RF exposure, which started on Gestation Day 5 of the dams (Fig. 2). The in vivo genotoxicity assays (Alkaline Comet Assay and Micronucleus Test) were performed at week 14 of the carcinogenicity study, corresponding to approximately 19 wk of total exposure from the start of maternal exposure. This timing was selected to ensure that the assays were performed after sufficient cumulative exposure to RF radiation and under steady-state exposure conditions, in accordance with OECD Test Guidelines (TG 489 and TG 474).

The Pig-a gene mutation assay was not conducted in the Japanese study because the method was not yet standardized or adopted under OECD Test Guidelines at the time of study initiation (2018). Instead, genotoxicity evaluation focused on the validated Alkaline Comet and Micronucleus assays, which were already established and internationally recognized.

The Alkaline Comet Assay was performed to assess DNA damage (Tice et al. 2000). Blood was collected from the abdominal vena cava under isoflurane anesthesia. Frontal cortex, hippocampus, cerebellum, liver, and peripheral blood leukocytes were collected and analyzed for the percentage of DNA in the tail (% tail DNA). The sample preparation and analysis for the Comet assay were outsourced to Trans Genic Inc. (Iwata, Shizuoka, Japan).

The Micronucleus Test was conducted to evaluate chromosomal damage. Bone marrow cells were collected from the left femur of the same animals used for the Comet assay. Smear preparations were fixed with methanol and stained with acridine orange fluorescent dye. A total of 4,000 immature erythrocytes were counted per animal to calculate the incidence of micronucleated immature erythrocytes (MNIEs%), and approximately 500 erythrocytes per animal were counted to determine the percentage of immature erythrocytes among all erythrocytes (IEs%). The sample preparation and analysis for the Micronucleus test were outsourced to SNBL INA Ltd (Ina, Nagano, Japan).

### Statistical analysis

Statistical analyses were performed to identify significant differences between groups (cage control vs. sham-exposed, cage control vs. RF-exposed, sham-exposed vs. RF-exposed) at significance levels of *P* < 0.05 or *P* < 0.01. For continuous data such as body weight, food consumption, body temperature, hematological and blood biochemical parameters, organ weights, %tail DNA, MNIEs%, and IEs%, F-tests were used for variance, followed by Student’s *t*-test (2-sided) for equal variances or Wilcoxon rank-sum test (2-sided) for unequal variances. For incidence data, including pathological findings (neoplastic and non-neoplastic) and littered/pregnant percentage, Fisher’s exact test (2-sided) was applied. Survival rates were analyzed using the Log-rank test and Kaplan-Meier method. Because statistically significant differences in survival rates were observed among the groups, the incidence of neoplastic lesions was analyzed using the Peto test. The Peto statistical test is widely used in carcinogenicity studies to compare tumor incidence among groups with differing survival rates (Peto 1974). It adjusts for survival differences, making it suitable for detecting treatment-related tumors without bias from differential mortality (Haseman 1984). This method is recommended by the NTP and OECD for evaluating neoplastic lesions in long-term studies. General condition and macroscopic necropsy findings were not subjected to statistical analysis. Statistical analyses were performed using Lab Site (Fujitsu Ltd Kanagawa, Japan), Stat Light 2000 (Yukms Co., Ltd, Tokyo, Japan), and SAS software (version 9.4, SAS Institute Inc., Cary, North Carolina).

## Results

### 28-Day preliminary study

**Fig. 3.**
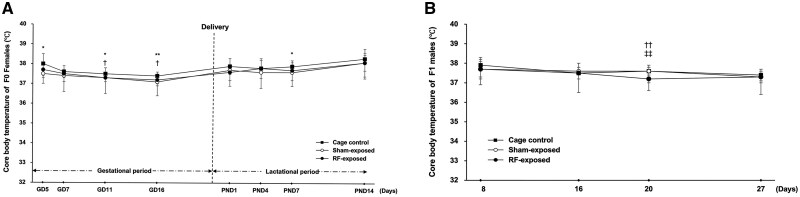
Core body temperature of the F0 females and the F1 males during the 28-d preliminary study. A) F0 females during the gestation and lactation periods. B) F1 males during the 28-d exposure period. Significant differences: *P < 0.05, **P < 0.01 cage control vs. sham exposed; ^†^*P* < 0.05, ^††^*P* < 0.01 cage control vs. RF-exposed; ^‡‡^*P* < 0.01 sham-exposed vs. RF exposed.

The 28-d preliminary study investigated the effects of 4 W/kg radiofrequency (RF) exposure on F0 and F1 rats. No effects of electromagnetic field (EMF) exposure were observed on various parameters indicative of reproductive performance, including gestation period, number of implantation sites, delivery index, live birth index, number of delivered per litter, number of live pups per litter, number of dead pups per litter, and sex ratio (data not shown). In F0 animals, body weight from postnatal day 14 to weaning and food consumption throughout the lactation period were significantly lower in the RF-exposed group compared with the sham-exposed group. Furthermore, after weaning, body weight remained significantly lower through day 14, and food consumption remained significantly lower or showed a decreasing trend until the end of the exposure period in the RF-exposed group compared with the sham-exposed group (Figs S1–S4). Core body temperature measurements for both F0 and F1 rats showed statistically significant variations among the cage control, sham-exposed, and RF-exposed groups (Fig. 3A and 3B). Analysis of hematological parameters revealed minor, yet statistically significant, differences in mean corpuscular volume (MCV), mean corpuscular hemoglobin (MCH), mean corpuscular hemoglobin concentration (MCHC), and reticulocytes (Table S1). Similarly, several clinical chemistry parameters, including blood urea nitrogen (BUN), total cholesterol (T-CHO), total protein (TP), albumin (ALB), albumin/globulin (A/G) ratio, inorganic phosphorus (IP), creatine phosphokinase (CK), and sodium (Na), exhibited statistically significant changes (Table S2). Furthermore, statistically significant differences were observed in both absolute and relative organ weights for the brain, liver, kidneys, and epididymides, as well as absolute lung weight (Tables S3 and S4). Despite these statistically significant changes in various parameters, comprehensive histopathological examination across a wide range of organs revealed no findings that were considered significant or adverse and attributable to RF exposure (Table S5). Although historical control data for Hsd:Sprague Dawley SD rats were not available at our facility because this strain was newly introduced from the United States for the NTP validation study, all observed changes in hematology, serum chemistry, and organ weights were small, lacked dose- or exposure-related trends, and were unsupported by any histopathological or organ-weight changes in target tissues (liver, kidney) and were not considered toxicologically significant.

### Results of 2-year carcinogenicity study

#### Reproductive findings, body weight, and food consumption until weaning

Reproductive findings, body weight and food consumption of F0 animals, and body weight of F1 offspring during the lactation period are presented in Table 1 and Fig. 5A–C. No significant differences were observed in gestation period, number of implantation sites, delivery index, live birth index, number of delivered per litter, number of live pups per litter, number of dead pups per litter, or sex ratio, indicating that RF exposure had no adverse effects on reproductive performance. In F0 animals, body weight during the lactation period and food consumption throughout the gestation and lactation periods were significantly lower or tended to be lower in the RF-exposed group compared with the cage control or sham-exposed groups. In addition, the body weight of F1 offspring during the lactation period was significantly lower in the RF-exposed group compared with the sham-exposed group.

#### Survival, body weight, food consumption, and clinical observation

**Fig. 4.**
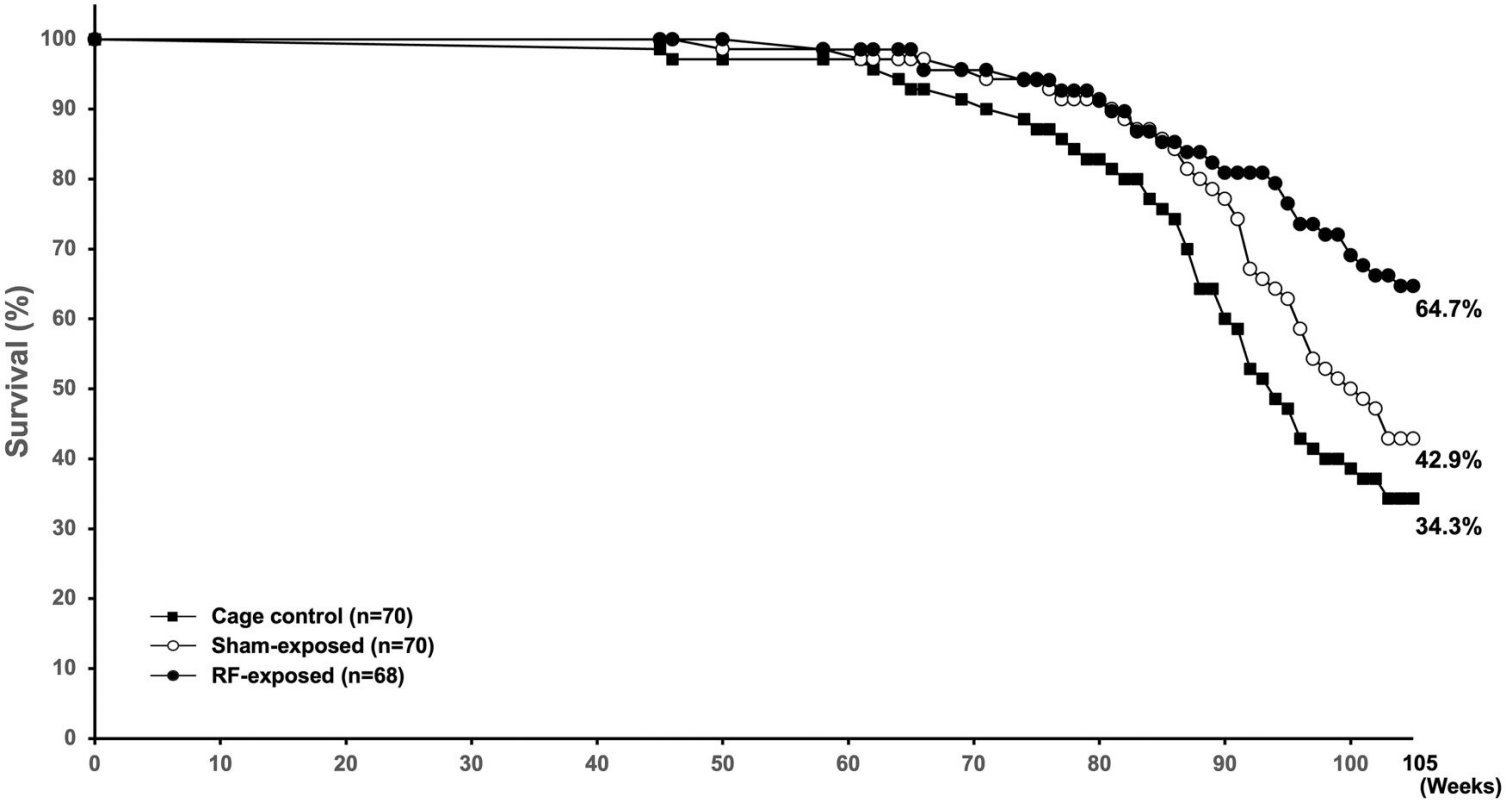
Kaplan-Meier Survival curves of the F1 males in the 2-yr carcinogenicity study.

The survival rates for the cage control, sham-exposed, and RF-exposed groups at 105 wk were 34.3% (24/70), 42.9% (30/70), and 64.7% (44/68), respectively. Two animals in the RF-exposed group that died at week 20 due to equipment malfunction were excluded from the analysis. An increase in mortality cases was observed in the sham-exposed group and cage control group from week 90 onwards, resulting in statistically significantly higher survival rates in the RF-exposed group at weeks 94, 100, and 105 (Fig. 4). Throughout the study, various clinical observations, such as abnormalities in posture, behavior, gait, and respiration, as well as skin or subcutaneous nodules/masses, were incidentally noted across groups. However, no changes attributable to electromagnetic exposure were observed in the general condition of the animals.

Body weight at the start of the carcinogenicity study was significantly higher in both the sham-exposed and RF-exposed groups compared with the cage control group. The RF-exposed group also showed a statistically significant lower body weight compared with the sham-exposed group at this time. This lower body weight in the RF-exposed group persisted, with significant differences from the sham-exposed group continuing until week 86. Compared with the cage control group, the RF-exposed group exhibited significantly lower body weights from week 4 to week 46, but then showed significantly higher body weights from week 86 until the end of the exposure period. The sham-exposed group also showed significantly higher body weights than the cage control group from week 22 until the end of the exposure period (Fig. 6).

Food consumption in the RF-exposed group was consistently and significantly lower than both the sham-exposed and cage control groups from the start of the carcinogenicity study until the end of the experiment, for the majority of the period. The sham-exposed group also showed sporadic, statistically significant lower food intake compared with the cage control group from week 3 to week 16, and significantly higher intake from week 96 until the end of the exposure (Fig. 7).

*Pathology Gross findings* at necropsy revealed no macroscopic pathological changes considered to be effects of electromagnetic exposure (data not shown). Splenic enlargement and various lymph node enlargements showed a higher trend in the RF-exposed group, which was attributed to the coincidental increase in Large Granular Lymphocytic (LGL) lymphoma incidence in this group. Conversely, a higher incidence of granular kidney surface (bilateral) was observed in the cage control and sham-exposed groups, reflecting the higher incidence of chronic progressive nephropathy in these groups.

*Neoplastic lesions* showed no significant differences in the incidence of benign tumors, malignant tumors, or total tumors among the groups. There was also no evidence of earlier tumor onset due to electromagnetic exposure (Table S6).

Specifically, for the *heart and brain*, which were areas of concern from previous studies:

- In the heart, endocardial schwannoma was observed in a small number of animals (1 case in the cage control, 1 in the sham-exposed, and 1 in the RF-exposed group), with no statistically significant differences among groups (Table 2).
- In the brain, benign granular cell tumor (3 cases in sham-exposed, 0 in cage control or RF-exposed), malignant granular cell tumor (1 case in cage control, 0 in sham-exposed or RF-exposed), and glioma, NOS (2 cases in cage control, 1 in RF-exposed, 0 in sham-exposed) were observed in a small number of animals (Table 2). Importantly, no statistically significant differences were found in the incidences of any of these brain tumors between the groups. Immunohistochemical analysis was performed on 2 cases of brain tumor, identified in the cage control group. Tumor cells were positive only for vimentin, whereas negative for GFAP, S100, and cytokeratin AE1/AE3 (Fig. 8).
- In the adrenal glands, an adenoma of the cortex was observed in 1 (1%) RF-exposed animal and in 3 (4%) sham-exposed animals, whereas no cases were found in the cage-control group. Benign pheochromocytomas were observed in 10 (14%) cage-control, 7 (10%) sham-exposed, and 9 (13%) RF-exposed animals. (Table 2). No statistically significant differences were noted in the incidences of these specific neoplastic lesions between the groups.

For other organs, various tumor types were observed across the groups; however, their incidences were generally comparable, with no statistically significant differences. Detailed data are provided in Table S6. An exception was LGL lymphoma, which showed a significantly higher incidence in the RF-exposed group (7 cases) compared with the sham-exposed group (1 case) using Fisher’s exact test (*P* = 0.0319). However, this difference was not significant when analyzed by Peto’s test (*P* = 1.00), suggesting it was an incidental finding likely influenced by the higher survival rate in the RF-exposed group. Conversely, adenocarcinoma of the jejunum was observed less frequently in the RF-exposed group (0 cases) compared with the sham-exposed group (6 cases), with a significant difference by Fisher’s exact test (*P* ≤ 0.05). This was also considered an incidental finding based on Peto’s test (*P* = 1.00).

*Non-neoplastic lesions* were observed in various organs and tissues, including heart, brain, and adrenal glands, across all groups (Table 3 and Table S7). Several non-neoplastic changes showed statistically significant differences in the RF-exposed group compared with the cage control or sham-exposed groups. These included an increase in foci of cellular alteration in the liver, mammary gland atrophy, testicular edema, atypical hyperplasia of the prostate, increased focal vacuolation of the adrenal cortex, and degeneration of nerve fibers in the trigeminal nerve. Among the non-neoplastic changes, only testicular edema showed statistically significant differences from both the cage control and sham-exposed groups. Because most affected animals were survivors (15/16, 23/24, and 49/50 in the cage control, sham-exposed, and RF-exposed groups, respectively), this finding is considered incidental rather than exposure-related. In addition to these findings, several observations showed significantly lower values in the exposure groups compared with the cage control or sham-exposed groups. Other non-neoplastic lesions were observed across all groups with comparable incidences and no statistically significant differences among them.

Detailed incidence data for all neoplastic and non-neoplastic lesions, as well as additional experimental data, are provided in the Supplementary Data, specifically in Table S6 (neoplastic lesions), Table S7 (non-neoplastic lesions), 28-d Supplementary Tables (Tables S1–S5), and 28-d Supplementary Figures (Figs S1–S4).

### Genotoxicity study

In the genotoxicity studies, the Alkaline Comet Assay (Table 4) assessed DNA damage in various tissues. The mean % tail DNA in the liver, peripheral blood, cerebellum, hippocampus, and frontal cortex did not show a statistically significant increase in either the sham-exposed or RF-exposed groups when compared with the cage control group. For example, in peripheral blood, the mean % tail DNA for cage control, sham-exposed, and RF-exposed groups were 0.81 ± 0.05, 0.79 ± 0.07, and 0.85 ± 0.06, respectively, indicating no significant differences. In contrast, the positive control group, which received ethyl methane sulfonate (200 mg/kg), consistently showed a statistically significant increase in % tail DNA across all examined organs, thus confirming the validity and sensitivity of the assay.

The Micronucleus test (Table 5) was performed to assess chromosomal damage. This test revealed no statistically significant increase in the frequency of micronucleated immature erythrocytes (MNIEs) in the bone marrow of male rats exposed to RF (mean 0.080% ± 0.041%) when compared with either the cage control (mean 0.080% ± 0.021%) or sham-exposed (mean 0.080% ± 0.033%) groups. The positive control group, however, exhibited a statistically significant increase in MNIEs (mean 0.540% ± 0.123%; *P < 0.05), demonstrating a clear positive response and confirming the assay’s sensitivity. Additionally, the immature erythrocyte (IE) percentages showed no significant differences between the test groups.

## Discussion

The present GLP-compliant 2-yr carcinogenicity and genotoxicity study demonstrated that long-term exposure of male Hsd:Sprague Dawley SD rats to 900 MHz CDMA-modulated RF-EMF at 4 W/kg did not produce evidence of exposure-related carcinogenic or genotoxic effects. The RF-exposed animals exhibited slightly lower body weight and food consumption but higher survival rates relative to the controls.

The key outcome of this study is the absence of statistically significant increases in neoplastic lesions in any of the examined organs, including the brain, heart, and adrenal glands (Table 2 and Table S6). Although glioma NOS in the brain and schwannoma in the heart were each observed in a single animal in the RF-exposed group, these incidences did not differ statistically from controls. These results contrast with the marginal increases in brain and heart tumors reported in the U.S. NTP study (Wyde et al. 2016; NTP 2018b). Importantly, the tumor incidences in our study are consistent with historical control data for Hsd:SD rats and fall within expected ranges (Charles River L 2012; NTP 2024). Although the number of animals per group (70 males) and the use of a single exposure level (4 W/kg) inevitably limited the statistical power compared with the multi-dose design of the NTP study, the scale and design of the present study were formulated with reference to the OECD Test Guideline 451 for carcinogenicity studies. The primary purpose of the present study was not to replicate the NTP findings quantitatively, but to evaluate the carcinogenic potential of long-term RF-EMF exposure under GLP-compliant and internationally harmonized bioassay conditions. Thus, our results should be interpreted in the context of a standard toxicological assessment designed to determine the presence or absence of carcinogenic activity rather than to detect rare tumor occurrences. Compared with the NTP study, which used multiple exposure levels and substantially larger group sizes, the statistical power to detect rare, low-incidence tumors is inherently lower in our design. Therefore, although no increases in such rare neoplastic findings were observed, very small changes in their occurrence cannot be completely ruled out.

One factor that may account for discrepancies with the NTP results is the survival rate. In our study, the survival of cage control rats was lower (34.3% at 105 wk) compared with RF-exposed rats (64.7%), potentially reducing the time window during which late-onset spontaneous tumors, such as gliomas in the brain or schwannomas in the heart, might manifest. Gliomas in SD rats are known to occur late in life, often after 90 wk of age (Weber et al. 2011; Bertrand et al. 2014; Sacaan et al. 2019), and their incidence is therefore heavily influenced by survival. This phenomenon has been extensively described in the literature and has led to recommendations for survival-adjusted statistical methods (eg, Poly-3, Poly-k, or Peto’s test) in long-term bioassays (Peto et al. 1980; Bailer and Portier 1988; Portier and Bailer 1989).

**Fig. 5.**
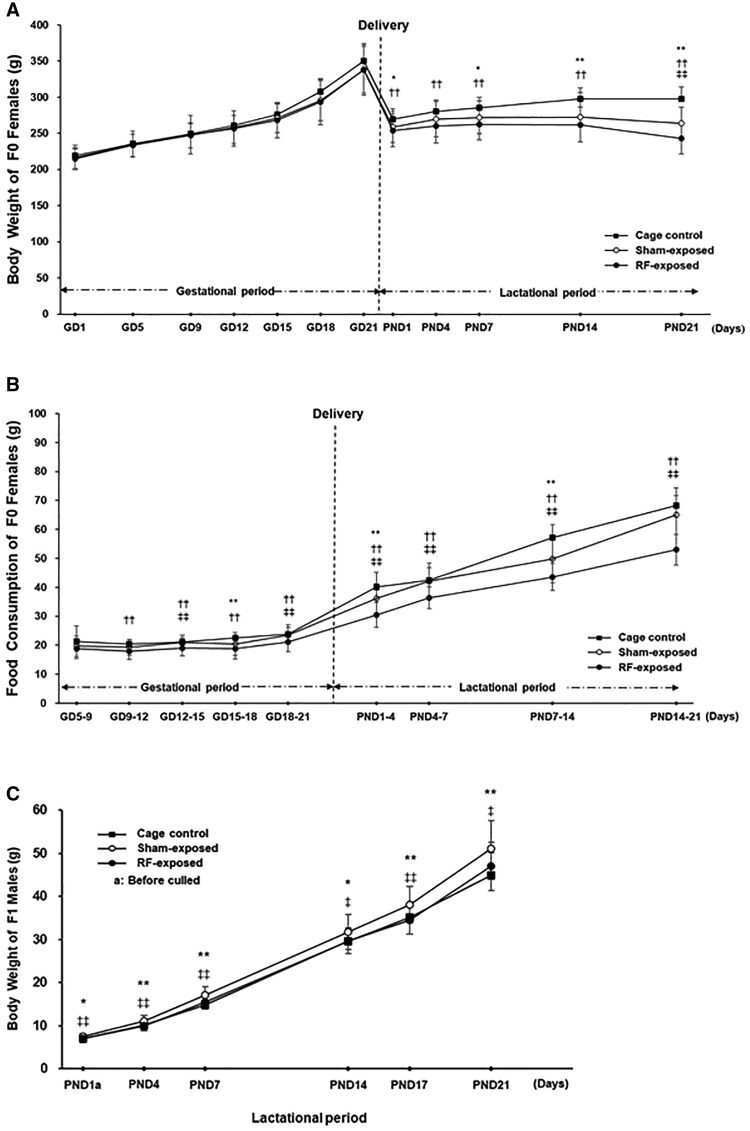
Body weight and food consumption in the F0 females, and body weight in the F1 males during the 2-yr carcinogenicity study. A) Body weight of the F0 females during the gestation and lactation periods. B) Food consumption of the F0 females during the gestation and lactation periods. C) Body weight of the F1 males during the lactation period. Significant differences: *P < 0.05, **P < 0.01 cage control vs. sham exposed; ^†^*P* < 0.05, ^††^*P* < 0.01 cage control vs. RF-exposed; ^‡‡^*P* < 0.01 sham-exposed vs. RF exposed.

**Fig. 6.**
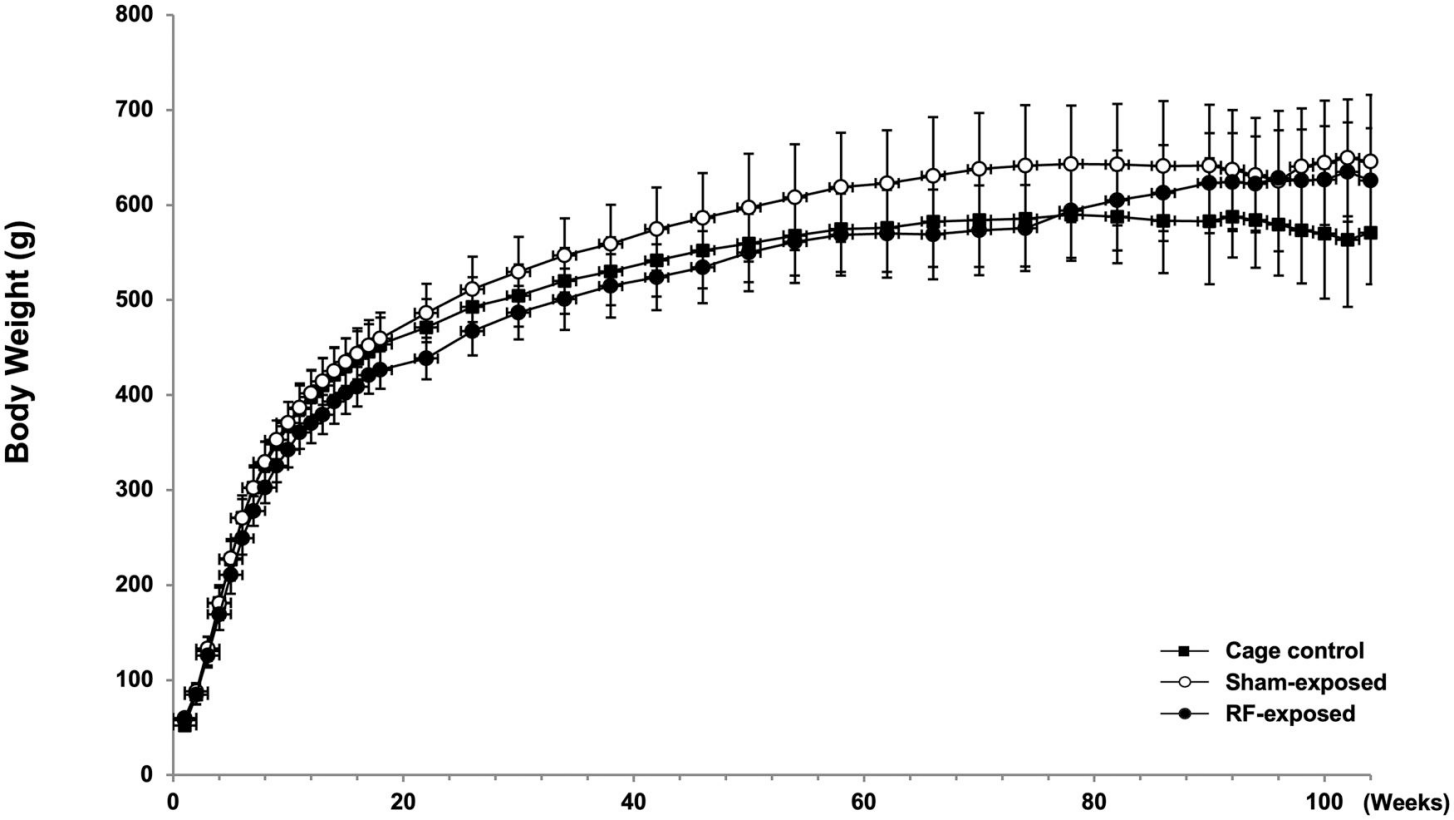
Body weight of the F1 males in the 2-yr carcinogenicity study.

**Fig. 7.**
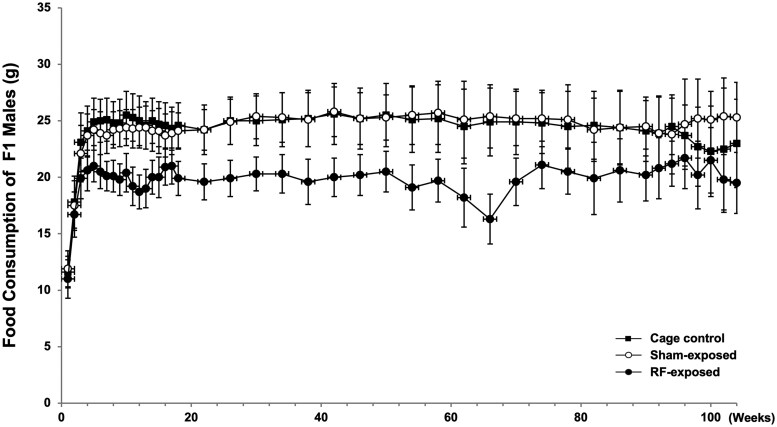
Food consumption of the F1 males in the 2-yr carcinogenicity study.

The survival rate in the sham and cage control groups was within the normal range and comparable with those reported in previous long-term studies, including the NTP bioassay. However, the RF-exposed group exhibited a slightly higher survival throughout the study period (Fig. 4). This difference may be partly explained by the consistently lower food consumption and body weight observed in the RF-exposed rats (Figs 5–7). Such conditions resemble mild caloric restriction, which has been reported to reduce age-related lesions such as chronic progressive nephropathy and cardiomyopathy, and to extend lifespan in rats (Keenan et al. 1995; Masoro 2005; Xu et al. 2015; Mitchell et al. 2019). Consistent with these reports, both lesions were significantly decreased and survival was correspondingly higher in the RF-exposed group in the present study. Accordingly, the relatively higher survival in the RF-exposed group may have been partly associated with reduced food intake rather than reflecting any direct biological effect of RF-EMF exposure. These observations are consistent with previous studies showing that moderate dietary restriction can extend lifespan and modulate tumor development in rats (Keenan et al. 1995; Turturro et al. 1999). Therefore, the slightly reduced body weight and food consumption in the RF-exposed group may have contributed to improved survival and could theoretically influence the background incidence of certain age-related neoplasms. However, such physiological modulation does not represent an exposure-related carcinogenic effect. In addition, the nominal difference in the incidence of large granular lymphocytic (LGL) lymphoma (*P* = 0.0319 by Fisher’s exact test) was not supported by Peto’s test (*P* = 1.00). As Haseman (1984) emphasized, *P* < 0.05 findings in rare tumors are often false positives, and a more stringent threshold of *P* < 0.01 is recommended. Moreover, LGL lymphoma is known to occur spontaneously and predominantly in aged rats as a late-onset neoplasm (Maronpot et al. 2016). In the present study, the RF-exposed group exhibited higher overall survival, resulting in somewhat more long-lived animals, which may have contributed to the apparent increase in LGL lymphoma incidence. These observations are best interpreted as incidental and without evidence of biological significance. The single case of glioma NOS observed in the RF-exposed group warrants discussion in terms of its toxicological and regulatory relevance. Gliomas in rats are relatively rare and poorly defined neuropathological entities. These lesions typically exhibit low proliferative activity, vague margins, and a lack of aggressive infiltration, distinguishing them from high-grade human gliomas such as glioblastoma multiforme (Koestner et al. 1999; Louis et al. 2016).

**Fig. 8.**
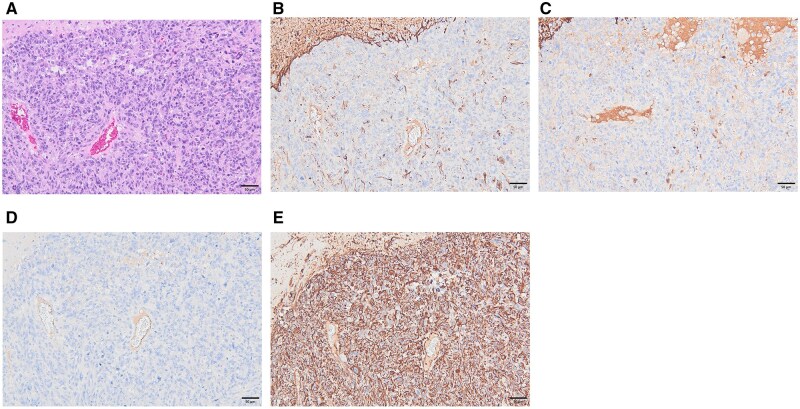
Immunohistochemical findings of Glioma, NOS observed in the cage control group. A) H&E staining (×200). B) Glial Fibrillary Acidic Protein (GFAP) staining (×200). C) S100 staining (×200). D) Cytokeratin (CK AE1/AE3) staining (×200). E) Vimentin staining (×200). The tumor showed strong positivity for vimentin, whereas GFAP, S100, and cytokeratin (CK AE1/AE3) were negative.

Several international pathology review panels, including those associated with the NTP and the Ramazzini Institute studies, have emphasized the ambiguous nature of rat gliomas and their limited utility for human cancer risk extrapolation (Lin 2022). Moreover, gliomas in rodents often lack hallmark molecular features seen in human gliomas, such as IDH1 mutations, 1p/19q co-deletions, or MGMT promoter methylation (Weber et al. 2011; Koelsche et al. 2015). The absence of these features suggests a fundamental biological difference between rodent and human gliomagenesis. Consequently, the U.S. Environmental Protection Agency (EPA) and other regulatory agencies have traditionally placed less weight on rodent gliomas in cancer risk assessments, unless supported by additional lines of evidence (US-EPA 2005; Cohen and Arnold 2011). In the present study, brain lesion was subjected to detailed histopathological evaluation, and diagnostic confirmation was performed using immunohistochemical markers (Fig. 8). The inclusion of immunohistochemistry thus strengthens the conclusion that the observed gliomas represent spontaneous background lesions rather than exposure-related neoplasms. Furthermore, immunohistochemical investigations by Kolenda-Roberts et al. (2013) demonstrated that many rat brain tumors historically diagnosed as astrocytomas are in fact malignant microglial tumors, whereas true astrocytomas are rarely observed in this species. This reclassification underscores the species-specific nature of rat glial tumors and further limits their relevance for extrapolating potential human health risks.

The notion of species-specificity is supported by comparative pathology data and cross-species carcinogenicity studies. For instance, gliomas have not been observed with increased frequency in mice exposed to RF-EMFs in similar experimental setups (Wyde et al. 2016; NTP 2018b), nor have epidemiological studies consistently demonstrated a causal relationship between RF exposure and glioma risk in humans (Austin 2002; INTERPHONE Study Group 2010; Frei et al. 2011; Little et al. 2012). Thus, although isolated gliomas in rats should be carefully documented, their toxicological relevance remains questionable unless accompanied by additional evidence of a dose-response relationship or mechanistic plausibility.

The same interpretive caution applies to the single case of endocardial schwannoma observed in the cage control group. Schwannomas are also late-onset lesions in rats and have a known background incidence in aged males, typically localized to the heart (Novilla et al. 1991). The NTP study observed increased schwannoma incidence in male rats at high RF exposure levels, but these findings were not reproduced in our study. The lack of any schwannoma cases in the RF-exposed group further weakens the hypothesized association between RF-EMFs and cardiac Schwann cell tumorigenesis.

Mechanistically, there is no well-established biological pathway linking RF-EMF exposure to Schwann cell transformation. RF radiation is non-ionizing and lacks sufficient energy to directly damage DNA or induce mutagenesis. Although thermal effects are possible at high SAR levels, our exposure system was carefully calibrated to avoid excessive temperature elevations, as supported by preliminary temperature monitoring and physiological data (Fig. 1) (Hirata and Fujiwara 2009).

Genotoxicity was assessed using the alkaline comet assay and the micronucleus test, in accordance with OECD Guidelines 489 and 474, respectively. In the present study, neither assay provided evidence of genotoxic effects from RF exposure.

These findings are consistent with prior genotoxicity studies that employed rigorous test guidelines and showed no RF-induced DNA damage in vivo (Herrala et al. 2018). Although several in vitro studies have reported genotoxic effects of RF-EMFs, particularly under conditions of high exposure duration, SAR, or temperature, these findings are inconsistent and not always replicated in in vivo systems (Diem et al. 2005; Zeni et al. 2005; Phillips et al. 2009). The current study adds to the body of negative evidence in the studies generated under GLP-compliant, guideline-based conditions, further supporting the conclusion that RF-EMFs do not exhibit genotoxic potential in mammalian systems.

A slight but consistent reduction in food consumption and body weight gain was observed in the RF-exposed rats compared with both sham-exposed and cage-control groups. These changes were statistically significant but not accompanied by any histopathological or biochemical alterations in the gastrointestinal tract or metabolic organs, indicating minor physiological variation rather than adverse toxicity.

Similar findings have been reported in some rat studies but not in other species such as mice or guinea pigs, suggesting possible species-specific sensitivity (Tsurita et al. 2000; Tas et al. 2014). In addition, recent work has discussed potential influences of radiofrequency fields on gut microbiota composition and metabolism (Luo et al. 2021). Although this mechanism was not examined here, it may partly explain inter-study variability in growth-related responses.

The findings of this study, taken together with prior animal bioassays and human epidemiological data, suggest that chronic RF exposure at 4 W/kg does not pose a significant carcinogenic or genotoxic risk. Although the NTP study raised concerns regarding rare tumors such as gliomas and schwannomas, our independent validation study found no consistent evidence supporting these associations.

Interpretation of rodent carcinogenicity studies must consider species-specific tumor biology, background incidence, and survival bias. The short lifespan of control animals may have limited the detection of rare spontaneous tumors, underscoring the need for survival-adjusted analyses and multiple lines of corroborating evidence before inferring human relevance.

The International Commission on Non-Ionizing Radiation Protection (ICNIRP) and the World Health Organization (WHO) recommend exposure limits for RF radiation based on thermal effects, noting that evidence for non-thermal carcinogenicity remains inconclusive. Our findings are consistent with this view, and the companion Korean study likewise detected no genotoxic or carcinogenic effects (ICNIRP 2020; WHO 2023).

In conclusion, this Japan–Korea collaborative effort provides a GLP-based independent verification of the NTP bioassay, indicating that, under harmonized 900 MHz CDMA exposure at 4 W/kg, we found no reproducible evidence of carcinogenic or genotoxic effects. The findings support the overall weight of evidence from previous RF safety evaluations (ICNIRP 2020; SCHEER (Scientific Committee on Health, Environmental and Emerging Risks) 2022), suggesting no reproducible carcinogenic potential of mobile phone–type RF exposure in rats.

## Supplementary Material

kfag002_Supplementary_Data

## Data Availability

All data generated or analyzed during this study are included in this published article and its supplementary information files. Additional raw data are available from the corresponding author upon reasonable request.
