# Supplementary material for: The International Collaborative Animal Study of mobile phone radiofrequency radiation carcinogenicity and genotoxicity: the Japanese study
Source: Toxicol Sci. 2026 Jan 12;209(3):kfag002. doi: 10.1093/toxsci/kfag002 (PMC13078595; doi:10.1093/toxsci/kfag002)
Supplement: kfag002_Supplementary_Data [file kfag002_supplementary_data.zip › toxsci-25-0642-File010.pdf]

Supplementary Tables

The International Collaborative Animal Study of Mobile Phone Radiofrequency Radiation Carcinogenicity and Genotoxicity: The Japanese Study

Katsumi Imaida[1*], Mayumi Kawabe[2,3,4], Jianqing Wang[5], Masanao Yokohira[4], Norio Imai[2,6], Kang-Hyun Han[7], Yong-Bum Kim[7], Sang Bong Jeon[8], Hye Sun Kim[9], and Young Hwan Ahn[9,10]

1 Kagawa University, Takamatsu, Kagawa 760-8521, Japan

2 DIMS Institute of Medical Science, Inc., Ichinomiya, Aichi 491-0113, Japan

3 Trans Genic Inc., Iwata, Shizuoka 437-1213, Japan

4 Faculty of Medicine, Kagawa University, Miki, Kagawa 761-0793, Japan

5 Department of Electrical and Mechanical Engineering, Nagoya Institute of Technology, Nagoya, Aichi 466-8555, Japan

6 Nihon Bioresearch Inc., Research Department, Hashima, Gifu 501-6251, Japan

7 Division of Next Generation Non-Clinical Research, Korea Institute of Toxicology, Daejeon 34114, Republic of Korea

8 Radio Research Division, Electronics and Telecommunications Research Institute (ETRI), Daejeon 34129, Republic of Korea

9 Department of Neurosurgery, Ajou University School of Medicine, Suwon 16499, Republic of Korea

10 Neuroscience Graduate Program, Department of Biomedical Sciences, Graduate School of Ajou University, Suwon 16499, Republic of Korea

Abbreviation

Hematology		
RBC	:	Total red blood cell count
HGB	:	Hemoglobin
HCT	:	Hematocrit
MCV	:	Mean corpuscular volume
MCH	:	Mean corpuscular hemoglobin
MCHC	:	Mean corpuscular hemoglobin concentration
PLT	:	Platelet count
WBC	:	Total leukocyte count
**Clinical chemistry**		
AST	:	Aspartate aminotransferase
ALT	:	Alanine aminotransferase
ALP	:	Alkaline phosphatase
γ-GTP	:	Gamma glutamyl transpeptidase
BUN	:	Blood urea nitrogen
CRE	:	Creatinine
GLU	:	Glucose
T-CHO	:	Total cholesterol
TG	:	Triglyceride
TP	:	Total protein
ALB	:	Albumin
A/G ratio	:	Albumin/globulin ratio
IP	:	Inorganic phosphorus
Ca	:	Calcium
CK	:	Creatine phosphokinase
Na	:	Sodium
K	:	Potassium
Cl	:	Chloride

Table S1. Hematology Data in 28-day Preliminary Study

	Cage control	Sham-exposed	RF-exposed
Level (W/kg)	0	0	4
No. of animals	10	8[a]	10
RBC (x10^4/μL)	656 ± 18	654 ± 16	655 ± 20
HGB (g/dL)	13.5 ± 0.4	13.2 ± 0.4	13.5 ± 0.4
HCT (%)	39.3 ± 1.1	39.2 ± 1.5	40.3 ± 1.0
MCV (fL)	60.0 ± 2.0	59.9 ± 1.4	61.5 ± 1.3 †
MCH (pg)	20.6 ± 0.6	20.2 ± 0.4	20.6 ± 0.3 †
MCHC (g/dL)	34.4 ± 0.7	33.7 ± 0.7	33.5 ± 0.4 **
PLT (x10^4/μL)	106.2 ± 14.0	106.2 ± 9.4	100.1 ± 7.8
Reticulocytes (x10^4/μL)	48.99 ± 6.48	57.10 ± 7.57 *	54.73 ± 4.94 *
Reticulocytes (%)	7.5 ± 1.0	8.7 ± 1.1 *	8.4 ± 0.7 *
WBC (x10^2/μL)	98.4 ± 17.4	93.3 ± 11.2	97.4 ± 14.8
**Leukocytes, differential (x10^2/μL)**			
Lymphocytes	89.6 ± 16.3	84.6 ± 11.4	88.5 ± 13.6
Neutrophils	5.8 ± 1.7	6.0 ± 1.3	6.1 ± 1.1
Eosinophils	0.7 ± 0.1	0.8 ± 0.2	0.7 ± 0.1
Basophils	0.0 ± 0.0	0.0 ± 0.0	0.0 ± 0.0
Monocytes	2.3 ± 0.6	1.9 ± 0.4	2.1 ± 0.7
**Leukocytes, differential (%)**			
Lymphocytes	91.0 ± 1.9	90.5 ± 2.1	90.8 ± 1.2
Neutrophils	5.9 ± 1.5	6.6 ± 1.8	6.3 ± 1.0
Eosinophils	0.7 ± 0.2	0.9 ± 0.2	0.8 ± 0.2
Basophils	0.0 ± 0.0	0.0 ± 0.0	0.0 ± 0.0
Monocytes	2.3 ± 0.6	2.1 ± 0.3	2.1 ± 0.4

Significantly different from the cage control group; *:p<0.05, **:p<0.01 (Student's t-test).

Significantly different from the sham-exposed group; †:p<0.05 (Student's t-test).

a: Data from two animals were omitted from the statistical analysis due to poor water intake caused by supplying trouble.

Table S2. Clinical Chemistry Data in 28-day Preliminary Study

	Cage control	Sham-exposed	RF-exposed
Level (W/kg)	0	0	4
No. of animals	10	8[a]	10
AST (U/L)	84 ± 18	129 ± 106	96 ± 12
ALT (U/L)	51 ± 5	75 ± 65	52 ± 6
ALP (U/L)	1240 ± 110	1316 ± 130	1253 ± 126
γ-GTP (U/L)	0.6 ± 0.1	0.6 ± 0.1	0.7 ± 0.1
BUN (mg/dL)	17.5 ± 1.9	14.5 ± 1.4 **	14.3 ± 0.9 **
CRE (mg/dL)	0.21 ± 0.01	0.21 ± 0.02	0.22 ± 0.01
GLU (mg/dL)	167 ± 28	172 ± 23	156 ± 16
T-CHO (mg/dL)	101 ± 5	88 ± 6 **	85 ± 8 **
TG (mg/dL)	111 ± 23	130 ± 28	113 ± 34
TP (g/dL)	5.3 ± 0.2	5.2 ± 0.1	5.4 ± 0.1 ††
ALB (g/dL)	3.9 ± 0.1	3.9 ± 0.1	4.1 ± 0.1 **, †
A/G ratio	2.79 ± 0.18	3.17 ± 0.19 **	3.13 ± 0.18 **
ACTH (pg/mL)	179.4 ± 47.5	180.9 ± 92.9	182.8 ± 107.9
IP (mg/dL)	9.2 ± 0.6	8.6 ± 0.4 *	8.8 ± 0.3
Ca (mg/dL)	11.0 ± 0.3	10.8 ± 0.2	10.9 ± 0.2
CK (U/L)	637 ± 269	745 ± 117	857 ± 159 *
Na (mmol/L)	141.8 ± 1.1	141.4 ± 1.3	140.9 ± 0.5 *
K (mmol/L)	5.69 ± 0.26	5.51 ± 0.30	5.55 ± 0.25
Cl (mmol/L)	100.5 ± 1.0	100.7 ± 1.9	101.0 ± 0.6

Significantly different from the cage control group; *:p<0.05, **:p<0.01 (Student's t-test or Wilcoxon rank-sum test).
Significantly different from the sham-exposed group; †:p<0.05, ††:p<0.01 (Student's t-test).
a: Data from two animals were omitted from the statistical analysis due to poor water intake caused by supplying trouble.

Table S3. Organ Weight Data in 28-day Preliminary Study

	Cage control	Sham-exposed	RF-exposed
Level (W/kg)	0	0	4
No. of animals	10	8[a]	10
Body weight (g)	262.8 ± 11.5	257.1 ± 7.2	250.1 ± 10.0 *
Brain (g)	1.770 ± 0.030	1.725 ± 0.040 *	1.733 ± 0.039 *
Heart (g)	0.974 ± 0.070	0.951 ± 0.060	0.915 ± 0.059
Lungs (g)	1.153 ± 0.056	1.118 ± 0.062	1.093 ± 0.070 *
Liver (g)	13.651 ± 0.634	12.926 ± 0.842	11.947 ± 1.058 **, †
Kidneys (g)	2.377 ± 0.067	2.188 ± 0.083 **	2.031 ± 0.130 **, ††
Spleen (g)	0.709 ± 0.048	0.655 ± 0.062	0.678 ± 0.035
Thymus (g)	0.685 ± 0.086	0.620 ± 0.069	0.626 ± 0.082
Pituitary gland (mg)	10.2 ± 0.9	10.2 ± 0.9	10.1 ± 0.6
Thyroid glands (mg)	20.6 ± 3.3	20.6 ± 3.1	18.6 ± 2.8
Adrenal glands (mg)	39.4 ± 3.4	40.6 ± 4.0	38.4 ± 2.0
Testes (g)	2.907 ± 0.124	2.886 ± 0.220	2.820 ± 0.218
Prostate (g)	0.565 ± 0.094	0.561 ± 0.042	0.536 ± 0.069
Epididymides (g)	0.389 ± 0.020	0.430 ± 0.037 **	0.398 ± 0.029
Seminal vesicles (g)	0.375 ± 0.058	0.404 ± 0.054	0.368 ± 0.061
Salivary glands	0.548 ± 0.043	0.530 ± 0.056	0.519 ± 0.023

Significantly different from the cage control group; *:p<0.05, **:p<0.01 (Student's t-test).

Significantly different from the sham-exposed group; †:p<0.05, ††:p<0.01 (Student's t-test).

a: Data from two animals were omitted from the statistical analysis due to poor water intake caused by supplying trouble.

Table S4. Relative Organ Weight (g/100g B.W.) Data in 28-day Preliminary Study

	Cage control	Sham-exposed	RF-exposed
Level (W/kg)	0	0	4
No. of animals	10	8[a]	10
Brain	0.675 ± 0.028	0.671 ± 0.015	0.693 ± 0.021 †
Heart	0.371 ± 0.022	0.370 ± 0.019	0.366 ± 0.016
Lungs	0.439 ± 0.021	0.435 ± 0.014	0.437 ± 0.024
Liver	5.195 ± 0.153	5.026 ± 0.258	4.771 ± 0.290 **
Kidneys	0.905 ± 0.036	0.851 ± 0.031 **	0.811 ± 0.028 **, †
Spleen	0.270 ± 0.015	0.255 ± 0.023	0.271 ± 0.018
Thymus	0.261 ± 0.035	0.241 ± 0.025	0.251 ± 0.033
Pituitary gland ($x10^3$)	3.90 ± 0.30	3.95 ± 0.36	4.03 ± 0.19
Thyroid glands ($x10^3$)	7.87 ± 1.37	8.01 ± 1.27	7.44 ± 1.08
Adrenal glands ($x10^3$)	15.02 ± 1.49	15.79 ± 1.57	15.36 ± 0.66
Testes	1.107 ± 0.049	1.123 ± 0.073	1.128 ± 0.075
Prostate	0.215 ± 0.036	0.218 ± 0.017	0.214 ± 0.026
Epididymides	0.148 ± 0.008	0.167 ± 0.014 **	0.159 ± 0.012 *
Seminal vesicles	0.143 ± 0.023	0.157 ± 0.020	0.147 ± 0.024
Salivary glands	0.209 ± 0.018	0.207 ± 0.023	0.208 ± 0.011

Significantly different from the cage control group; *:p<0.05, **:p<0.01 (Student's t-test).

Significantly different from the sham-exposed group; †:p<0.05 (Student's t-test).

a: Data from two animals were omitted from the statistical analysis due to poor water intake caused by supplying trouble.

Table S5. Histopathological Findings in 28-day Preliminary Study

Organs / Findings	Group / Level (W/kg) / Number of animals	Cage control	Sham-exposed	RF-exposed
	Group	Cage control	Sham-exposed	RF-exposed
	Level (W/kg)	0	0	4
	Number of animals	10	8[a]	10
**CARDIOVASCULAR SYSTEM**				
Heart		(10) b	(8)	(10)
Aorta		(10)	(8)	(10)
**HEMATOPOIETIC SYSTEM**				
Bone marrow		(10)	(8)	(10)
Spleen		(10)	(8)	(10)
Mesenteric lymph node		(10)	(8)	(10)
Mandibular lymph node		(10)	(8)	(10)
Thymus		(10)	(8)	(10)
**RESPIRATORY SYSTEM**				
Lung/bronchial		(10)	(8)	(10)
Trachea		(10)	(8)	(10)
Larynx		(10)	(8)	(10)
Infiltrate, Inflammatory cell, minimal		1	0	0
Nasal cavity		(10)	(8)	(10)
**DIGESTIVE SYSTEM**				
Esophagus		(10)	(8)	(10)
Stomach		(10)	(8)	(10)
Erosion, Glandular, minimal		0	1	0
Pancreas		(10)	(8)	(10)
Duodenum		(10)	(8)	(10)
Jejunum		(10)	(8)	(10)
Ileum		(10)	(8)	(10)
Inflammation, minimal		1	0	0
Cecum		(10)	(8)	(10)
Colon		(10)	(8)	(10)
Rectum		(10)	(8)	(10)
Liver		(10)	(8)	(10)
Salivary gland		(10)	(8)	(10)
Pharynx		(10)	(8)	(10)
Peyer patch		(10)	(8)	(10)
**URINARY SYSTEM**				
Kidney		(10)	(8)	(10)
Cast, Hyaline, minimal		3	0	1
Cyst, minimal		0	0	1
Interstitial nephritis, minimal		0	1	0
Mineralization, Pelvis, minimal		1	0	0
Urinary bladder		(10)	(8)	(10)
**REPRODUCTIVE SYSTEM**				
Testis		(10)	(8)	(10)
Degeneration, Tubular, minimal		1	0	0
Epididymis		(10)	(8)	(10)
Reduced sperm, Luminal, minimal		1	0	0
Prostate		(10)	(8)	(10)
Seminal vesicle		(10)	(8)	(10)
Preputial gland		(10)	(8)	(10)
Infiltrate, Lymphocyte , minimal		1	2	5
slight		1	2	0
**ENDOCRINE SYSTEM**				
Pituitary gland		(10)	(8)	(10)
Persistent Rathke's pouch		4	2	2
Thyroid glands		(10)	(8)	(10)
Ectopic tissue, Thymus		4	0	1
Parathyroid glands		(10)	(7)	(10)
Adrenal glands		(10)	(8)	(10)
**NERVOUS SYSTEM**				
Brain		(10)	(8)	(10)
Spinal cord		(10)	(8)	(10)
Sciatic nerve		(10)	(8)	(10)
**SPECIAL SENSE ORGANS**				
Eye		(10)	(8)	(10)
Harderian gland		(10)	(8)	(10)
**INTEGUMENTARY SYSTEM**				
Skin/subcutis		(10)	(8)	(10)
Mammary gland		(10)	(8)	(10)
**MUSCULOSKELETAL SYSTEM**				
Skeletal muscle		(10)	(8)	(10)
Femur		(10)	(8)	(10)
Sternum		(10)	(8)	(10)

a: Data from two animals were omitted from the statistical analysis due to poor water intake caused by supplying trouble.
b: Number in parentheses indicates the number of animals examined.
Not significant between groups.

Table S6. Summary of the Incidence of Neoplastic Lesions in 2-year Carcinogenicity Study

Organs / Findings	Level (W/kg)	Cage control 0 (%)	Sham-exposed 0 (%)	RF-exposed 4 (%)
**HEMATOPOIETIC SYSTEM**				
Spleen		[70] a	[70]	[68]
	Hemangioma	1 (1)	0	0
Lymph nodes[b]		[11]	[8]	[7]
	Hemangioma	0	1 (13)	0
Mesenteric lymph node		[70]	[70]	[68]
	Hemangioma	1 (1)	0	1 (1)
	Hemangiosarcoma	1 (1)	1 (1)	2 (3)
Thymus		[68]	[70]	[68]
	Thymoma, benign	1 (1)	3 (4)	2 (3)
**RESPIRATORY SYSTEM**				
Lungs		[70]	[70]	[68]
	Adenoma, bronchiolo-	2 (3)	0	1 (1)
	Adenocarcinoma	0	1 (1)	0
Nasal cavity		[70]	[70]	[67]
	Adenoma	0	1 (1)	0
**DIGESTIVE SYSTEM**				
Forestomach		[70]	[70]	[68]
	Papilloma, squamous cell	2 (3)	0	0
	Carcinoma, squamous cell	0	1 (1)	0
Pancreas (exocrine)		[70]	[70]	[67]
	Adenoma, acinar cell	4 (6)	5 (7)	3 (4)
Duodenum		[70]	[70]	[67]
	Adenocarcinoma	2 (3)	0	0
Jejunum		[70]	[68]	[65]
	Adenocarcinoma	1 (1)	6 (9)	0
Liver		[70]	[70]	[68]
	Adenoma, hepatocyte	1 (1)	0	1 (1)
	Carcinoma, hepatocyte	3 (4)	1 (1)	0
**URINARY SYSTEM**				
Kidneys		[70]	[70]	[68]
	Adenoma	1 (1)	0	0
	Carcinoma	0	1 (1)	0
	Nephroblastoma	1 (1)	0	0
	Hemangiosarcoma	0	0	2 (3)
**REPRODUCTIVE SYSTEM**				
Testes		[70]	[70]	[68]
	Adenoma, leydig cell	1 (1)	0	0
Prostate		[70]	[70]	[68]
	Adenoma	4 (6)	7 (10)	5 (7)
Preputial gland		[70]	[70]	[67]
	Papilloma, squamous cell	1 (1)	0	0
**ENDOCRINE SYSTEM**				
Pituitary gland		[70]	[70]	[67]
	Adenoma, pars distalis	9 (13)	14 (20)	14 (21)
	Adenoma, pars intermedia	1 (1)	2 (3)	0
	Carcinoma, pars distalis	1 (1)	1 (1)	0
Thyroid glands		[70]	[70]	[68]
	Adenoma, C-cell	8 (11)	15 (21)	16 (24)
	Adenoma, follicular cell	0	0	2 (3)
	Carcinoma, C-cell	1 (1)	1 (1)	4 (6)
Parathyroid glands		[70]	[69]	[61]
	Adenoma	0	1 (1)	0
Pancreas (endocrine)		[70]	[70]	[67]
	Adenoma, islet cell	3 (4)	5 (7)	4 (6)
	Carcinoma, islet cell	5 (7)	7 (10)	3 (4)

**NERVOUS SYSTEM**			
Trigeminal nerve	[70]	[70]	[67]
Sarcoma, NOS	1 (1)	0	0
Schwannoma, malignant	1 (1)	1 (1)	0
**INTEGUMENTARY SYSTEM**			
Skin	[70]	[70]	[68]
Adenoma, sebaceous cell	1 (1)	0	0
Keratoacanthoma	2 (3)	4 (6)	2 (3)
Papilloma, squamous cell	2 (3)	1 (1)	0
Tumor, hair follicle, benign	7 (10)	4 (6)	6 (9)
Carcinoma, squamous cell	0	1 (1)	0
Tumor, basal cell, malignant	1 (1)	0	0
Subcutaneous tissue[b]	[8]	[15]	[6]
Fibroma	2 (25)	8 (53)	3 (50)
Lipoma	0	2 (13)	1 (17)
Fibrosarcoma	2 (25)	2 (13)	0
Mammary gland	[70]	[69]	[68]
Fibroadenoma	0	1 (1)	1 (1)
**MUSCULOSKELETAL SYSTEM**			
Maxilla[b]	[1]	[0]	[0]
Sarcoma, NOS	1 (100)	-	-
Sternum	[70]	[70]	[68]
Chondroma	0	1 (1)	0
**OTHERS**			
Systemic neoplasm	[70]	[70]	[68]
Lymphoma, LGL	3 (4)	1 (1)	7 (10)
Leukemia, erythroid	0	0	1 (1)
Leukemia, myeloid	0	2 (3)	0
Sarcoma, histiocytic	1 (1)	1 (1)	3 (4)
Thoracic cavity[b]	[1]	[0]	[0]
Schwannoma, malignant	1 (100)	-	-
Abdominal cavity[b]	[1]	[5]	[2]
Mesothelioma, malignant	0	2 (40)	0
Schwannoma, malignant	1 (100)	3 (60)	1 (50)
Zymbal's gland[b]	[0]	[1]	[1]
Carcinoma, sebaceous cell	-	1 (100)	0
Carcinosarcoma	-	0	1 (100)
Tail[b]	[2]	[0]	[0]
Keratoacanthoma	1 (50)	-	-
**NEOPLASTIC SUMMARY**			
Number of tumors			
Benign	65	88	72
Malignant	36	36	28
Total	101	124	100
Number of animals with tumors			
Benign	40	49	42
Malignant	30	32	24
Single	22	22	22
Multiple	31	36	32
Total	53	58	54

a: Number in square brackets indicates the number of animals examined.
b: Only organs bearing macroscopically detected tumors were subjected to histological evaluation.
No statistically significant differences in any tumor incidence were observed among the groups by Peto's test.

Table S7. Summary of the Incidence of Non-Neoplastic Lesions in 2-year Carcinogenicity Study

Organs / Findings	Level (W/kg)	Cage control 0 (%)	Sham-exposed 0 (%)	RF-exposed 4 (%)
**CARDIOVASCULAR SYSTEM**				
Aorta		[70] a	[70]	[68]
	Mineralization	0	0	1 (1)
**HEMATOPOIETIC SYSTEM**				
Bone marrow		[70]	[70]	[68]
	Atrophy, focal	1 (1)	0	0
	Hyperplasia, adipocyte	7 (10)	4 (6)	6 (9)
	Hyperplasia, bone marrow	14 (20)	12 (17)	4 (6)
Spleen		[70]	[70]	[68]
	Congestion	0	0	1 (1)
	Hemorrhage	1 (1)	0	0
	Atrophy	4 (6)	5 (7)	5 (7)
	Pigment, macrophage	10 (14)	7 (10)	6 (9)
	Infiltrate, inflammatory cell	1 (1)	0	0
	Polyarteritis	5 (7)	3 (4)	0
	Extramedullary hematopoiesis, increased	11 (16)	8 (11)	5 (7)
	Hyperplasia, white pulp	0	1 (1)	0
Lymph nodes[b]		[11]	[8]	[7]
	Erythrocytes, intrasinusoidal	7 (64)	5 (63)	0
	Dilatation, sinus	1 (10)	0	0
	Aggregates, increased, macrophage	2 (18)	0	0
	Hyperplasia, angiomatous	1 (10)	0	0
	Hyperplasia, mast cell	2 (18)	0	0
	Hyperplasia, lymphoid	0	0	1 (14)
Mesenteric lymph node		[70]	[70]	[68]
	Erythrocyte, intrasinusoidal	6 (9)	3 (4)	1 (1)
	Dilatation, sinus	2 (3)	7 (10)	2 (3)
	Pigment, macrophage	4 (6)	8 (11)	11 (16)
	Aggregates, increased, macrophage	3 (4)	3 (4)	5 (7)
	Infiltrate, neutrophilic	2 (3)	0	0
	Inflammation, granulomatous	0	0	1 (1)
	Polyarteritis	1 (1)	2 (3)	0
	Extramedullary hematopoiesis	0	0	1 (1)
	Hyperplasia, angiomatous	2 (3)	1 (1)	0
	Hyperplasia, lymphoid	3 (4)	4 (6)	4 (6)
Mandibular lymph node		[70]	[70]	[68]
	Erythrocytes, intrasinusoidal	1 (1)	1 (1)	0
	Atrophy, lymphoid	2 (3)	0	0
	Dilatation, sinus	9 (13)	7 (10)	9 (13)
	Infiltrate, inflammatory cell	2 (3)	0	0
	Hyperplasia, mast cell	1 (1)	0	0
	Hyperplasia, lymphoid	1 (1)	1 (1)	0
	Hyperplasia, plasma cell	0	0	1 (1)
Thymus		[68]	[70]	[68]
	Hemorrhage	0	0	2 (3)
	Cyst, epithelial	5 (7)	6 (9)	3 (4)
	Involution	64 (94)	68 (97)	56 (82)
	Polyarteritis	16 (24)	9 (13)	0
	Hyperplasia, epithelium	23 (34)	9 (13)	4 (6)
	Hyperplasia, lymphoid	0	2 (3)	1 (1)
**RESPIRATORY SYSTEM**				
Lungs		[70]	[70]	[68]
	Edema	2 (3)	0	0
	Hemorrhage	2 (3)	2 (3)	1 (1)
	Thrombus	1 (1)	1 (1)	0
	Mineralization	0	1 (1)	1 (1)
	Infiltrate, inflammatory cell	8 (11)	16 (23)	5 (7)
	Inflammation	12 (17)	3 (4)	1 (1)
	Polyarteritis	1 (1)	0	0
	Macrophage, increased	16 (23)	12 (17)	18 (26)
	Hyperplasia, bronchiolo-alveolar	0	2 (3)	0

Trachea		[70]	[70]	[68]
Larynx		[70]	[70]	[68]
	Infiltrate, inflammatory cell	1 (1)	0	2 (3)
	Inflammation	2 (3)	2 (3)	5 (7)
	Hyperplasia, respiratory epithelium	0	0	1 (1)
Nasal cavity		[70]	[70]	[67]
	Fibrous osteodystrophy	16 (23)	10 (14)	1 (1)
	Infiltrate, inflammatory cell, lumen	9 (13)	7 (10)	12 (18)
	Inflammation	14 (20)	13 (19)	9 (13)
	Polyarteritis	6 (9)	0	0
	Hyperplasia, olfactory epithelium	1 (1)	0	1 (1)
	Hyperplasia, respiratory epithelium	23 (33)	31 (44)	28 (42)
**DIGESTIVE SYSTEM**				
Esophagus		[70]	[70]	[68]
	Erosion/ulcer	1 (1)	0	0
Forestomach		[70]	[70]	[68]
	Erosion/ulcer	2 (3)	3 (4)	1 (1)
	Infiltrate, inflammatory cell	2 (3)	0	1 (1)
	Hyperplasia, basal cell	0	2 (3)	0
	Hyperplasia, squamous cell	3 (4)	4 (6)	1 (1)
Glandular stomach		[70]	[70]	[68]
	Erosion/ulcer	6 (9)	9 (13)	7 (10)
	Mineralization	5 (7)	5 (7)	1 (1)
	Necrosis, mucosa	1 (1)	0	0
	Infiltrate, inflammatory cell	1 (1)	2 (3)	1 (1)
	Polyarteritis	5 (7)	3 (4)	0
Pancreas (exocrine)		[70]	[70]	[67]
	Hemorrhage	0	0	1 (1)
	Thrombus	1 (1)	0	0
	Atrophy, acinar cell	9 (13)	7 (10)	4 (6)
	Inflammation	1 (1)	0	1 (1)
	Polyarteritis	29 (41)	21 (30)	1 (1)
	Hyperplasia, acinar cell	17 (24)	17 (24)	15 (22)
	Focus, basophilic	2 (3)	0	4 (6)
Duodenum		[70]	[70]	[67]
	Dilatation, crypt	1 (1)	6 (9)	2 (3)
	Dysplastic crypt, Peyer’s patch	1 (1)	0	0
	Erosion/ulcer	2 (3)	2 (3)	0
Jejunum		[70]	[68]	[65]
	Dysplastic crypt, Peyer’s patch	30 (43)	24 (35)	28 (43)
	Polyarteritis	1 (1)	0	0
Ileum		[67]	[62]	[61]
	Dysplastic crypt, Peyer’s patch	8 (12)	5 (8)	10 (16)
	Polyarteritis	2 (3)	1 (2)	0
Cecum		[69]	[64]	[65]
	Erosion/ulcer	4 (6)	3 (5)	0
	Polyarteritis	12 (17)	10 (16)	0
Colon		[70]	[70]	[65]
	Dysplastic crypt, Peyer’s patch	3 (4)	3 (4)	5 (8)
	Erosion/ulcer	1 (1)	0	0
	Polyarteritis	9 (13)	9 (13)	1 (2)
Rectum		[70]	[70]	[67]
	Erosion/ulcer	1 (1)	0	0
	Metaplasia, squamous cell	1 (1)	0	0
	Polyarteritis	3 (4)	0	0
Liver		[70]	[70]	[68]
	Angiectasis	1 (1)	1 (1)	0
	Cyst, bile duct	1 (1)	2 (3)	0
	Degeneration, cystic	8 (11)	7 (10)	10 (15)
	Fatty change	5 (7)	5 (7)	5 (7)
	Necrosis	9 (13)	3 (4)	4 (6)
	Infiltrate, inflammatory cell	0	0	1 (1)
	Polyarteritis	9 (13)	8 (11)	0
	Cholangiofibrosis	0	0	1 (1)
	Extramedullary hematopoiesis	4 (6)	4 (6)	1 (1)
	Focus of cellular alteration	40 (57)	49 (70)	51 (75)
	Hyperplasia, bile duct	10 (14)	14 (20)	1 (1)

Mandibular glands		[70]	[70]	[68]
	Atrophy	8 (11)	4 (6)	1 (1)
	Mineralization, media/wall artery	2 (3)	2 (3)	1 (1)
	Necrosis	0	0	1 (1)
	Hypertrophy, acinar cell	5 (7)	1 (1)	4 (6)
Sublingual glands		[70]	[70]	[68]
	Atrophy	5 (7)	2 (3)	0
	Infiltrate, inflammatory cell	1 (1)	0	1 (1)
	Hypertrophy, acinar cell	0	1 (1)	0
Pharynx		[70]	[70]	[68]
	Inflammation	0	2 (3)	0
Peyer's patch		[69]	[70]	[67]
	Hyperplasia, lymphoid	1 (1)	3 (4)	0
Mesenterium[b]		[3]	[0]	[1]
	Inflammation, lipogranulomatous	0	-	1 (100)
	Polyarteritis	3 (100)	-	0
**URINARY SYSTEM**				
Kidneys		[70]	[70]	[68]
	Hemorrhage	0	0	1 (1)
	Infarct	1 (1)	0	0
	Thrombus	1 (1)	0	0
	Accumulation, hyaline droplets	1 (1)	1 (1)	2 (3)
	Cyst	2 (3)	0	0
	Mineralization	3 (4)	2 (3)	1 (1)
	Necrosis, papilla	0	0	1 (1)
	Chronic Progressive Nephropathy	69 (99)	67 (96)	54 (79)
	Nephropathy, obstructive	0	0	1 (1)
	Pyelonephritis	1 (1)	1 (1)	1 (1)
	Dilatation, pelvis	0	1 (1)	1 (1)
	Hyperplasia, tubule	0	1 (1)	2 (3)
Urinary bladder		[70]	[69]	[68]
	Hemorrhage	0	1 (1)	0
	Infiltrate, inflammatory cell	2 (3)	0	1 (1)
	Hyperplasia, urothelium	1 (1)	1 (1)	0
**REPRODUCTIVE SYSTEM**				
Testes		[70]	[70]	[68]
	Edema	16 (23)	24 (34)	50 (74)
	Atrophy, tubule	21 (30)	26 (37)	15 (22)
	Spermatocele	3 (4)	1 (1)	1 (1)
	Sperm stasis	10 (14)	7 (10)	4 (6)
	Polyarteritis	39 (56)	32 (46)	1 (1)
	Sperm granuloma	4 (6)	5 (7)	1 (1)
	Hyperplasia, leydig cell	1 (1)	3 (4)	3 (4)
Epididymides		[70]	[70]	[68]
	Atrophy, duct	27 (39)	26 (37)	7 (10)
	Infiltrate, inflammatory cell	3 (4)	1 (1)	1 (1)
	Polyarteritis	13 (19)	8 (11)	0
Prostate		[70]	[70]	[68]
	Hemorrhage	0	1 (1)	0
	Atrophy	7 (10)	3 (4)	0
	Mineralization	1 (1)	0	0
	Infiltrate, inflammatory cell	9 (13)	8 (11)	4 (6)
	Inflammation	9 (13)	13 (19)	7 (10)
	Polyarteritis	2 (3)	1 (1)	0
	Hyperplasia, atypical	3 (4)	16 (23)	13 (19)
Seminal vesicles		[70]	[70]	[68]
	Atrophy	20 (29)	14 (20)	2 (3)
	Inflammation	0	1 (1)	1 (1)
	Hyperplasia, atypica	0	3 (4)	2 (3)
Preputial gland		[70]	[70]	[67]
	Atrophy	3 (4)	1 (1)	1 (1)
	Dilatation	11 (16)	12 (17)	10 (15)
	Infiltrate, inflammatory cell	10 (14)	10 (14)	7 (10)
	Inflammation	37 (53)	36 (51)	46 (69)

**ENDOCRINE SYSTEM**			
Pituitary gland	[70]	[70]	[67]
Hemorrhage	0	1 (1)	0
Cyst	8 (11)	5 (7)	10 (15)
Aberrant craniopharyngeal structure	1 (1)	0	0
Gliosis, pars nervosa	0	1 (1)	0
Hyperplasia, pars distalis	22 (31)	31 (44)	28 (42)
Hyperplasia, pars intermedia	0	2 (3)	3 (4)
Thyroid glands	[70]	[70]	[68]
Vacuolation, C-cell	0	0	1 (1)
Polyarteritis	3 (4)	3 (4)	0
Fibrosis	0	0	1 (1)
Hyperplasia, C-cell	12 (17)	15 (21)	13 (19)
Hyperplasia, follicular cell	1 (1)	2 (3)	0
Parathyroid glands	[70]	[69]	[61]
Hyperplasia, focal	2 (3)	1 (1)	0
Hyperplasia, diffuse	54 (77)	45 (65)	1 (2)
Pancreas (endocrine)	[70]	[70]	[67]
Inflammation	0	0	1 (1)
Hyperplasia, islet cell	3 (4)	7 (10)	4 (6)
**NERVOUS SYSTEM**			
Spinal cord	[70]	[70]	[68]
Radiculoneuropathy	44 (63)	50 (71)	53 (78)
Sciatic nerve	[70]	[70]	[68]
Cholesterol clefts	0	5 (7)	0
Degeneration, nerve fiber	6 (9)	8 (11)	2 (3)
Mineralization, media/wall artery	4 (6)	2 (3)	3 (4)
Trigeminal nerve	[70]	[70]	[67]
Degeneration, nerve fiber	48 (69)	55 (79)	57 (85)
**SPECIAL SENSE SYSTEM**			
Eyes	[70]	[70]	[68]
Atrophy, outer retina	5 (7)	1 (1)	0
Degeneration, lens fiber	4 (6)	1 (1)	2 (3)
Retinal rosettes	1 (1)	0	0
Mineralization, cornea	1 (1)	1 (1)	0
Infiltrate, inflammatory cell, cornea	7 (10)	8 (11)	2 (3)
Inflammation	6 (9)	7 (10)	3 (4)
Harderian glands	[70]	[70]	[68]
Atrophy	8 (11)	8 (11)	7 (10)
Dilatation	1 (1)	0	0
Infiltrate, inflammatory cell	2 (3)	0	1 (1)
Inflammation	0	0	1 (1)
Polyarteritis	1 (1)	0	0
Hyperplasia, acinar cell	8 (11)	12 (17)	5 (7)
**INTEGUMENTARY SYSTEM**			
Skin	[70]	[70]	[68]
Edema, dermis	2 (3)	0	0
Erosion/ulcer	1 (1)	1 (1)	1 (1)
Infiltrate, inflammatory cell	1 (1)	0	0
Inflammation, adnexa	1 (1)	1 (1)	0
Cyst, squamous	2 (3)	0	2 (3)
Hyperplasia, epidermis	1 (1)	0	0
Subcutaneous tissue[b]	[8]	[15]	[6]
Infiltrate, inflammatory cell	1 (13)	0	0
Inflammation	0	1 (7)	2 (33)
Polyarteritis	2 (25)	1 (7)	0
Fibrosis	0	0	1 (17)
Mammary gland	[70]	[69]	[68]
Atrophy	6 (9)	3 (4)	11 (16)
Dilatation	4 (6)	6 (9)	5 (7)
Infiltrate, inflammatory cell	1 (1)	0	0
Hyperplasia, lobuloalveolar	1 (1)	0	2 (3)
Tail[b]	[2]	[0]	[0]
Inflammation	1 (50)	-	-
Hyperkeratosis	1 (50)	-	-
Hyperplasia, epidermis	1 (50)	-	-

**MUSCULOSKELETAL SYSTEM**				
Skeletal muscle		[70]	[70]	[68]
	Atrophy	11 (16)	9 (13)	4 (6)
	Infiltrate, inflammatory cell	3 (4)	1 (1)	1 (1)
Femur		[70]	[70]	[68]
	Cyst, bone	12 (17)	8 (11)	7 (10)
	Fibrous osteodystrophy	17 (24)	11 (16)	1 (1)
	Bone, increased, trabeculae	0	1 (1)	1 (1)
Sternum		[70]	[70]	[68]
	Fibrous osteodystrophy	17 (24)	11 (16)	2 (3)
	Bone, increased, trabeculae	0	1 (1)	0
**OTHERS**				
Adipose tissue[b]		[1]	[5]	[5]
	Fat necrosis	1 (100)	2 (40)	3 (60)
	Inflammation	0	1 (20)	1 (20)
	Hyperplasia, adipose tissue	0	1 (20)	0

a: Number in square brackets indicates the number of animals examined.
b: Only organs bearing macroscopically detected tumors were subjected to histological evaluation.
