# Supplementary material for: The International Collaborative Animal Study of mobile phone radiofrequency radiation carcinogenicity and genotoxicity: the Japanese study
Source: Toxicol Sci. 2026 Jan 12;209(3):kfag002. doi: 10.1093/toxsci/kfag002 (PMC13078595; doi:10.1093/toxsci/kfag002)

Supplementary Figures

The International Collaborative Animal Study of Mobile Phone Radiofrequency Radiation Carcinogenicity and Genotoxicity: The Japanese Study

Katsumi Imaida1*, Mayumi Kawabe2,3,4, Jianqing Wang5, Masanao Yokohira4, Norio Imai2,6, Kang-Hyun Han7, Yong-Bum Kim7, Sang Bong Jeon8, Hye Sun Kim9, and Young Hwan Ahn9,10

1 Kagawa University, Takamatsu, Kagawa 760-8521, Japan

2 DIMS Institute of Medical Science, Inc., Ichinomiya, Aichi 491-0113, Japan

3 Trans Genic Inc., Iwata, Shizuoka 437-1213, Japan

4 Faculty of Medicine, Kagawa University, Miki, Kagawa 761-0793, Japan

5 Department of Electrical and Mechanical Engineering, Nagoya Institute of Technology, Nagoya, Aichi 466-8555, Japan

6 Nihon Bioresearch Inc., Research Department, Hashima, Gifu 501-6251, Japan

7 Division of Next Generation Non-Clinical Research, Korea Institute of Toxicology, Daejeon 34114, Republic of Korea

8 Radio Research Division, Electronics and Telecommunications Research Institute (ETRI), Daejeon 34129, Republic of Korea

9 Department of Neurosurgery, Ajou University School of Medicine, Suwon 16499, Republic of Korea

10 Neuroscience Graduate Program, Department of Biomedical Sciences, Graduate School of Ajou University, Suwon 16499, Republic of Korea

Figure S1. Body Weight Data of F0 in 28-day Preliminary Study

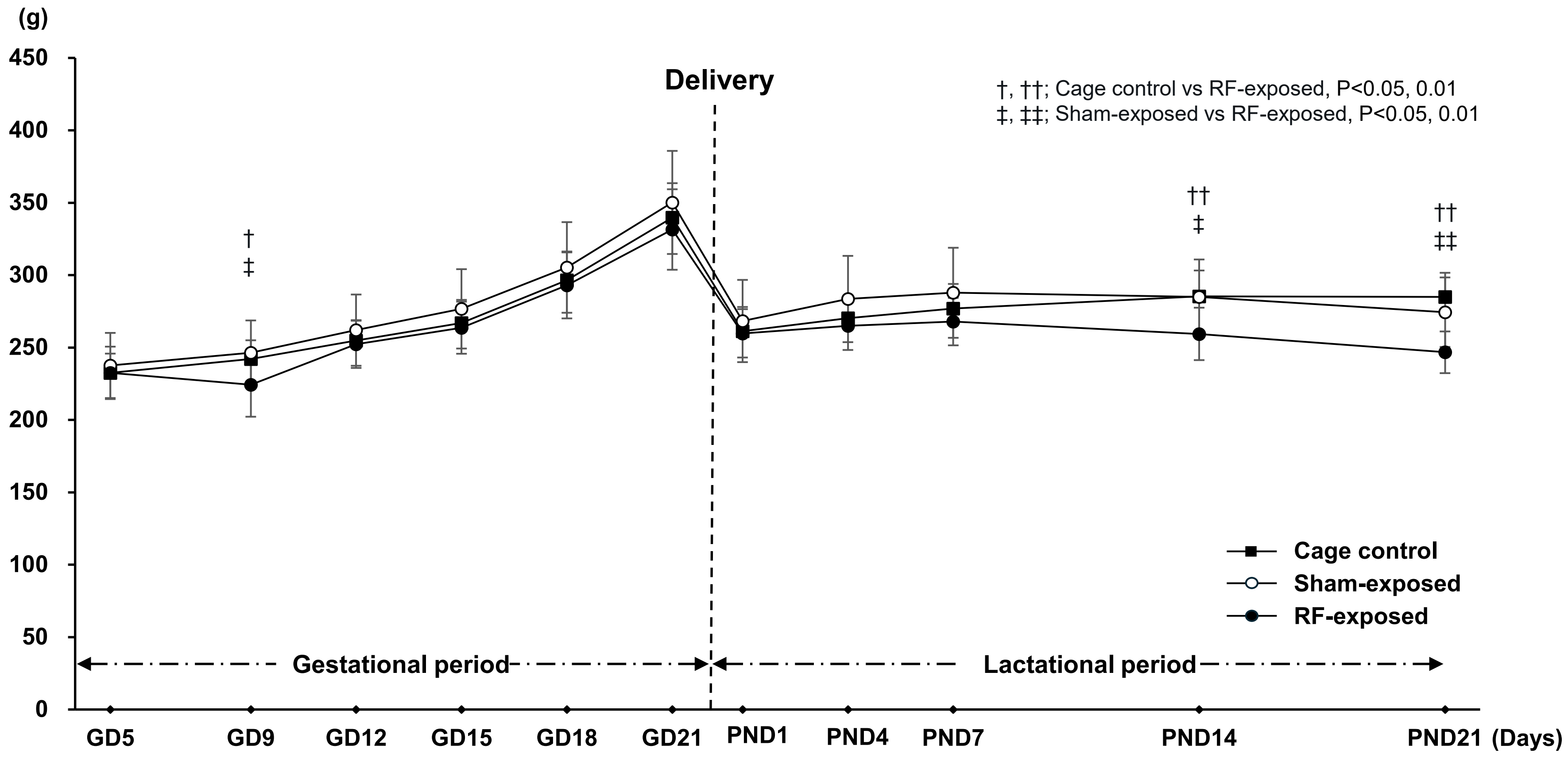

Figure S2. Food Consumption Data of F0 in 28-day Preliminary Study

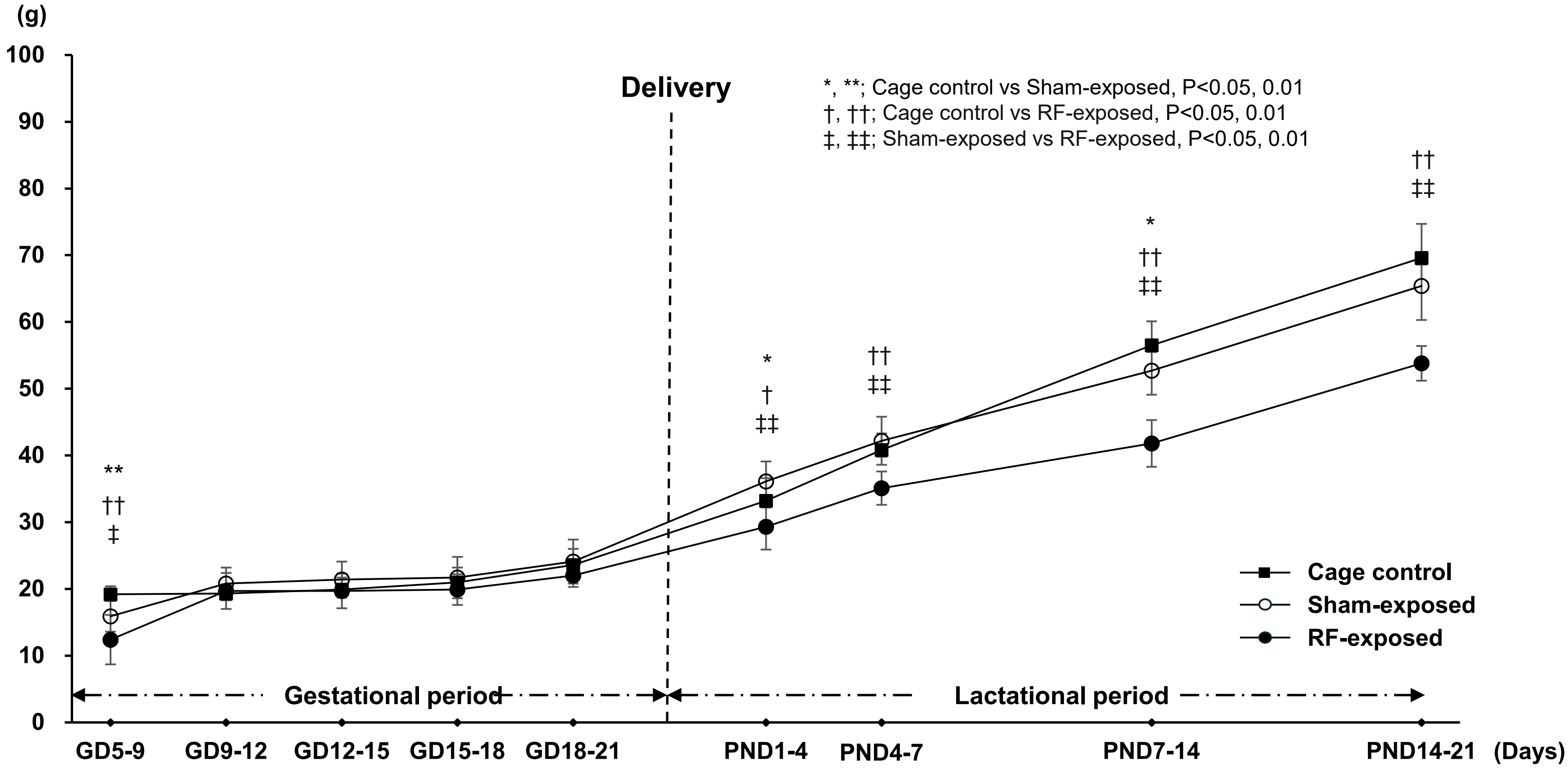

Figure S3. Body Weight Data of F1 in 28-day Preliminary Study

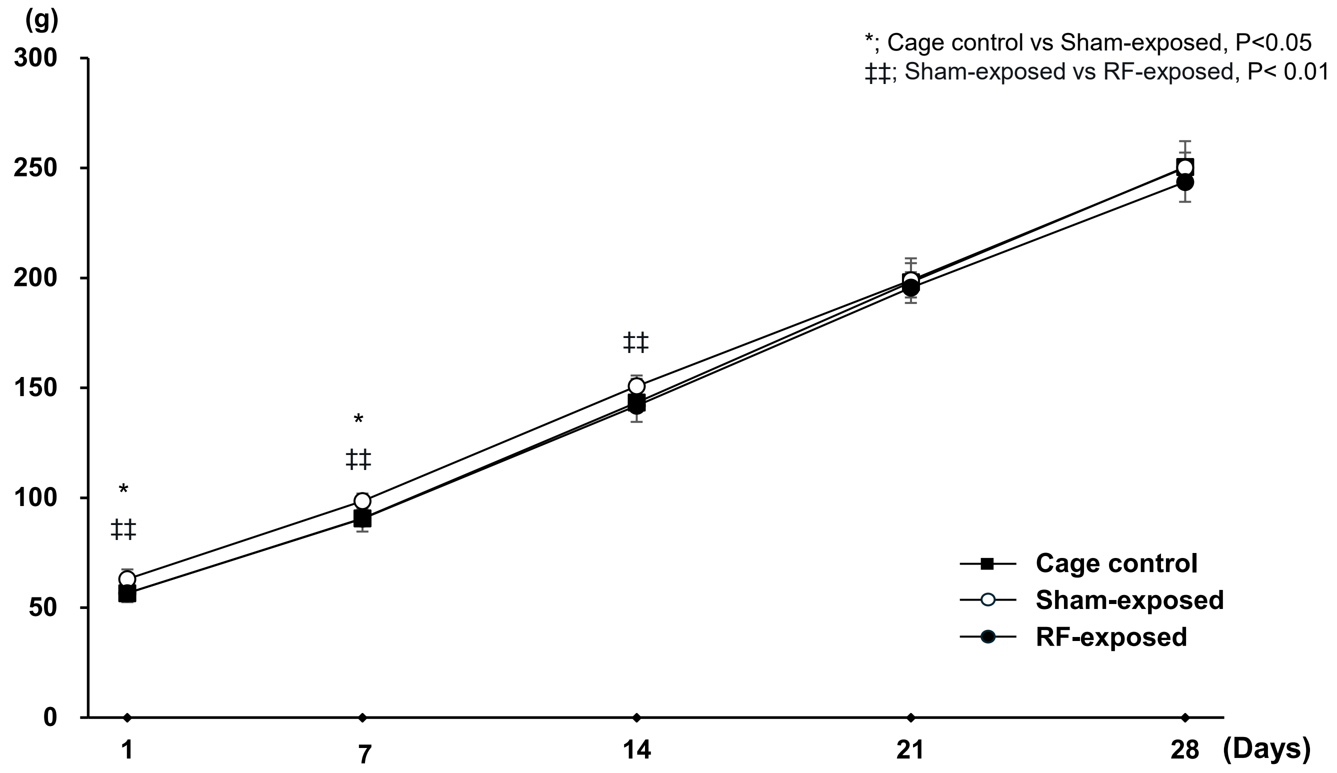

Figure S4. Food Consumption Data of F1 in 28-day Preliminary Study

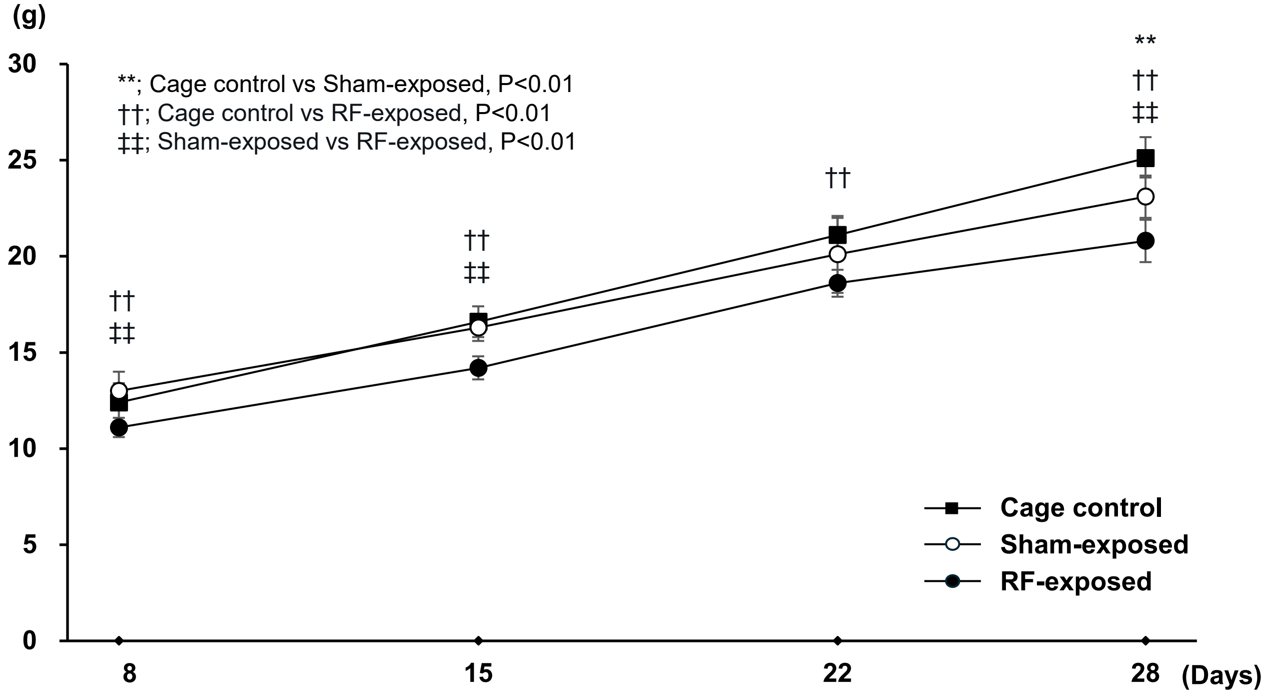

Supplement: kfag002_Supplementary_Data [file kfag002_supplementary_data.zip › toxsci-25-0642-File011.pdf]
